# Adipocytes Promote Cisplatin Resistance through Secreting A1BG and Regulating NAMPT/PARP1 Axis‐Mediated DNA Repair in Osteosarcoma

**DOI:** 10.1002/advs.202502926

**Published:** 2025-06-25

**Authors:** Yonghui Liang, Zhaohui Li, Lina Tang, Zhen Pan, Xiang Fei, Chen Tan, Aina He, Qingcheng Yang, Dongdong Cheng

**Affiliations:** ^1^ Department of Orthopedic Surgery Shanghai Sixth People's Hospital Affiliated to Shanghai Jiao Tong University School of Medicine Shanghai 200233 China; ^2^ Department of Oncology Shanghai Sixth People's Hospital Affiliated to Shanghai Jiao Tong University School of Medicine Shanghai 200233 China; ^3^ Institute of Clinical Chemistry and Laboratory Medicine, Section Mass Spectrometry and Proteomics University Medical Center Hamburg‐Eppendorf (UKE) 20246 Hamburg Germany

**Keywords:** adipocytes, cisplatin resistance, dna repair, osteosarcoma

## Abstract

Obesity is increasingly recognized as a negative prognostic factor for cancers, including osteosarcoma. However, the mechanisms linking obesity to chemoresistance in osteosarcoma remain unclear. This study found obesity is significantly associated with poor responses to cisplatin‐based chemotherapy in osteosarcoma patients. In vitro, adipocyte‐conditioned medium (Adi‐CM) induced cisplatin resistance, while peritumoral adipocytes and diet‐induced obesity (DIO) reduce the cisplatin efficacy in vivo. Mechanistically, Adi‐CM enhanced DNA repair by the PARP1/ATM pathway activation. Proteomic analysis identified A1BG, a secreted protein upregulated in adipocytes from chemoresistant patients, as a key mediator of this effect. A1BG depletion in adipocytes restored cisplatin sensitivity, whereas recombinant A1BG enhanced resistance and promoted DNA repair. Further investigation revealed a direct interaction between A1BG and NAMPT, leading to the stabilization of NAMPT and an increased NAD^+^ production. This enhanced PARP1 activity and subsequent DNA repair. Importantly, pharmacological inhibition of NAMPT and PARP1 using FK886 and Olaparib, respectively, reversed Adi‐CM‐induced cisplatin resistance and restored cisplatin sensitivity in osteosarcoma cells, DIO mouse models, and patient‐derived organoids. A novel link between obesity and cisplatin resistance in osteosarcoma is established, highlighting the A1BG/NAMPT/PARP1 axis as a critical driver. Targeting this axis may represent a promising therapeutic strategy for overcoming obesity‐associated chemoresistance in osteosarcoma.

## Graphical abstract

The proposed working model for how adipocytes participate in cisplatin resistance in osteosarcoma. The adipocytes secret A1BG and stabilize NAMPT, leading to increased NAD⁺ levels and subsequent increased PARP1‐mediated DNA repair, resulting in cisplatin resistance in osteosarcoma finally.

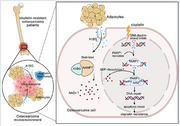

## 1 Introduction

Osteosarcoma, the most common primary malignant bone tumor in children and adolescents,^[^ ^1^ ^]^ has a 70% long‐term survival rate due to advancements in neoadjuvant chemotherapy since the 1970s. However, this survival rate has plateaued in recent decades, primarily due to chemoresistance.^[^ ^2^ ^]^ Chemoresistant osteosarcoma is associated with increased lung metastasis, tumor recurrence, and poor prognosis.^[^ ^3^ ^]^ Although the mechanisms underlying chemoresistance in osteosarcoma remain unclear, emerging evidence suggests that obesity is a contributing factor.^[^ ^4^ ^]^ Obesity, characterized by adipocyte accumulation, can impair drug delivery and release paracrine factors that negatively affect therapeutic outcomes.^[^ ^4^, ^5^ ^]^ Several studies have proposed a link between adipocytes and poor responses to both chemotherapy^[^ ^6^ ^]^ and radiotherapy.^[^ ^7^ ^]^ However, the specific role of adipocytes in osteosarcoma chemotherapy, particularly cisplatin resistance, remains poorly understood.

Cisplatin, a cornerstone of osteosarcoma chemotherapy, exerts anti‐tumor effects by forming DNA intra‐strand crosslinks, impairing DNA replication, inducing DNA double‐strand breaks (DSBs), and promoting apoptosis.^[^ ^8^ ^]^ Resistance to cisplatin presents a significant clinical challenge and can result in poor patient outcomes.^[^ ^9^ ^]^ Several mechanisms contribute to cisplatin resistance in osteosarcoma, including reduced oxidative stress,^[^ ^10^ ^]^ altered drug transporter activity,^[^ ^11^ ^]^ and enhanced DNA repair capacity.^[^ ^12^ ^]^ Notably, increased DNA repair, particularly through nicotinamide phosphoribosyltransferase (NAMPT)‐mediated NAD^+^ biosynthesis and the DNA damage response (DDR) pathway involving PARP1, plays a crucial role in cisplatin resistance.^[^ ^13^ ^]^ Upon recruitment to DNA lesions, PARP1 utilizes NAD^+^ to catalyze poly(ADP‐ribose) formation, thereby activating the DNA repair process.^[^ ^14^ ^]^ Both NAMPT^[^ ^15^ ^]^ and PARP1^[^ ^16^ ^]^ have been implicated in cisplatin resistance; however, their precise roles in osteosarcoma require further investigation.

In this study, we examined the effect of adipocytes on cisplatin resistance in osteosarcoma. Our findings revealed a negative correlation between high body mass index (BMI) and chemotherapeutic response in patients with osteosarcoma. Additionally, we demonstrated that adipocytes directly promote cisplatin resistance in osteosarcoma cells, likely by activating DNA repair pathways. Proteomic analysis identified A1BG, a secreted glycoprotein overexpressed in various cancers,^[^ ^17^ ^]^ as a potential mediator of adipocyte‐induced cisplatin resistance. Mechanistically, the results show that A1BG interacts with NAMPT to enhance NAD^+^ levels, leading to increased PARP1‐mediated ADP ribosylation and subsequent DNA repair. Our study provides novel evidence that adipocytes contribute to cisplatin resistance in osteosarcoma by activating the A1BG/NAMPT/PARP1 axis. This finding suggests a potential therapeutic target for overcoming chemoresistance in obese patients with osteosarcoma.

## 2 Result

### 2.1 Adipocytes Enhance Cisplatin Resistance in Osteosarcoma

**Figure 1:**
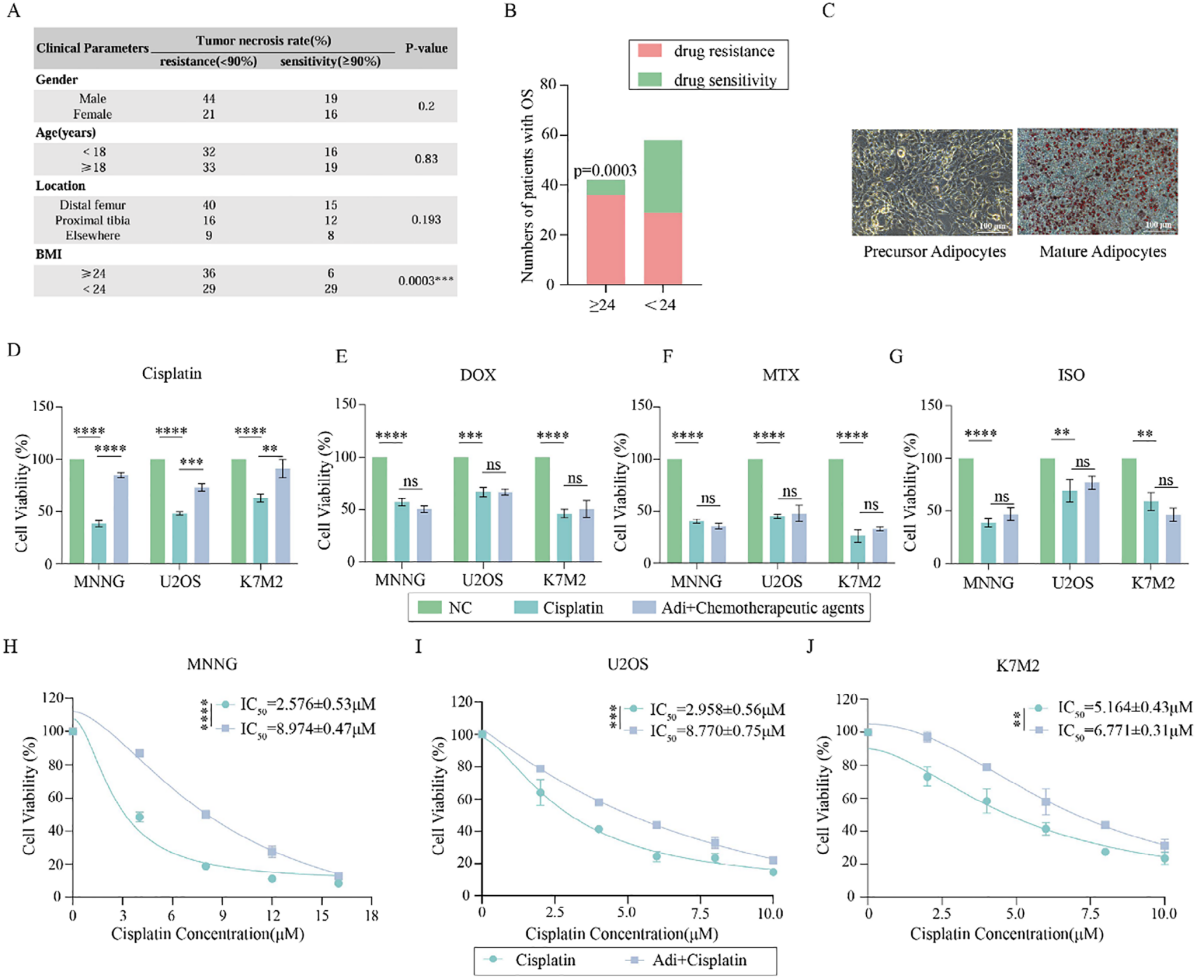
Adipocytes enhance Cisplatin Resistance in Osteosarcoma. A) Correlation analysis of tumor necrosis rate (%) with clinical variables in 100 patients with osteosarcoma. B) Correlation analysis between tumor necrosis rate (%) and BMI. C) Oil Red O staining of precursor adipocytes and mature adipocytes. D–G) CCK‐8 assay assessing osteosarcoma cell viability (MNNG, U2OS, K7M2) following treatment with cisplatin(6 µM), doxorubicin(400 nM), methotrexate(300 µM), or ifosfamide(300 µM) under control or Adi‐CM conditions (n = 3). H–J) Osteosarcoma cells (MNNG, U2OS, K7M2) were treated with cisplatin at varying concentrations for 48 h under control or Adi‐CM conditions, and the IC_50_ was calculated (n = 3). **p < 0.01; ***p < 0.001; ****p < 0.0001.

Clinical evidence increasingly suggests that obesity adversely affects the prognosis of patients with osteosarcoma, with chemoresistance being a major factor contributing to poor clinical outcomes.^[^ ^4a^ ^]^ To elucidate the relationship between obesity and therapeutic resistance in osteosarcoma, we analyzed patient data, including BMI and tumor necrosis rate, with BMI adjusted for age in adolescent patients.^[^ ^18^ ^]^ All patients received the complete standard chemotherapy regimen for osteosarcoma, known as MAPI, which involves the sequential administration of high‐dose methotrexate, doxorubicin, cisplatin, and ifosfamide. Our clinical analysis revealed a statistically significant inverse correlation between BMI and tumor necrosis rate, indicating that a higher BMI was associated with a reduced tumor response to chemotherapy (**Figure** **1**A,B). In contrast, no significant associations were observed between tumor necrosis rates and other clinical variables, such as sex, age, or tumor site (Figure 1A).

To determine the stage at which obesity influences resistance to chemotherapy during the MAPI regimen, we performed a series of in vitro experiments. First, we differentiated 3T3‐L1 precursor adipocytes into mature adipocytes using previously established protocols.^[^ ^19^ ^]^ Successful differentiation was confirmed within 15 days by the accumulation of lipid droplets (Figure 1C) and the upregulation of adipocyte‐specific markers, including FABP4 and leptin (Figure S1A–C, Supporting Information). To investigate the impact of adipocytes on the chemotherapy response, we collected conditioned medium from mature adipocytes (Adi‐CM) and treated osteosarcoma cell lines (MNNG, U2OS, and K7M2) with various chemotherapeutic agents for 48 h. Notably, Adi‐CM significantly enhanced the viability of osteosarcoma cells exposed to cisplatin (Figure 1D), whereas no such effect was observed after treatment with doxorubicin, methotrexate, or ifosfamide (Figure 1E–G). Cisplatin resistance was further investigated in osteosarcoma cells treated with cisplatin in combination with human‐derived adipocyte‐conditioned medium (Figure S2, Supporting Information).

To more precisely quantify the influence of Adi‐CM on cisplatin resistance, we measured the half‐maximal inhibitory concentration (IC_50_) of cisplatin in these cell lines under control and Adi‐CM conditions. In the absence of Adi‐CM, the IC_50_ values for cisplatin were 2.576, 2.958, and 5.164 µM in MNNG, U2OS, and K7M2 cells, respectively. Strikingly, the presence of Adi‐CM significantly increased the IC_50_ values to 8.974, 8.770, and 6.771 µM, respectively (Figure 1H–J). Importantly, this adipocyte‐mediated resistance was not limited to cisplatin but extended to other platinum‐based chemotherapeutics, including carboplatin and oxaliplatin (Figure S3A,B, Supporting Information). Collectively, these findings demonstrate that adipocytes profoundly enhance cisplatin resistance in osteosarcoma cells, potentially extending to other platinum‐based drugs.

### 2.2 Adipocytes Promote Cisplatin Resistance in Osteosarcoma by Enhancing DNA Repair

**Figure 2:**
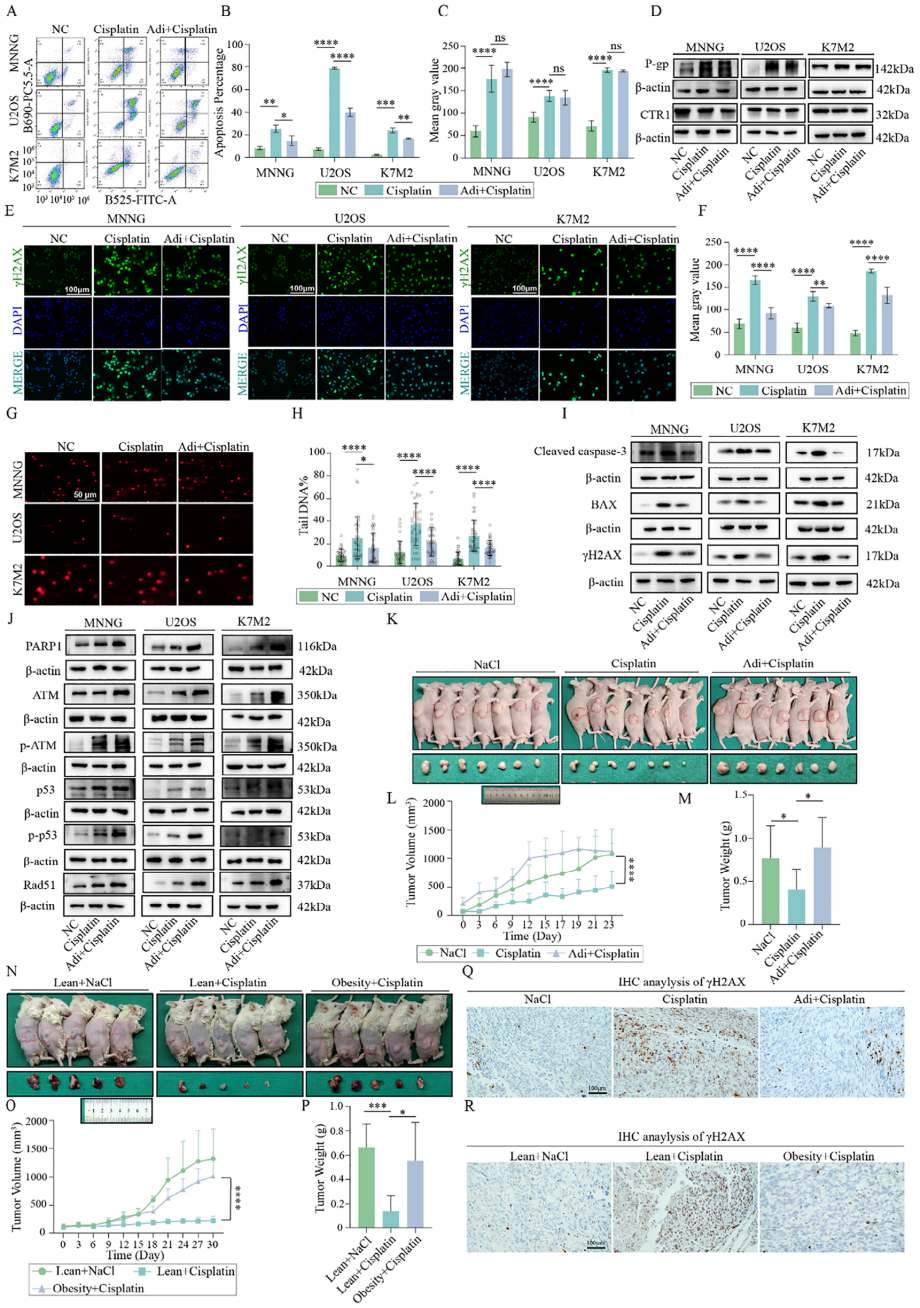
Adipocytes Promote Cisplatin Resistance in Osteosarcoma by Enhancing DNA Repair. A,B) Apoptosis assay of osteosarcoma cells (MNNG, U2OS, K7M2) treated with cisplatin under control or Adi‐CM conditions. C) The quantitative analysis of ROS levels (n = 3). D) Western blot analysis of P‐gp and CTR1 protein in osteosarcoma cells. E,F) Immunofluorescence analysis of γH2AX induction in osteosarcoma cells (MNNG, U2OS, K7M2) following cisplatin treatment under control or Adi‐CM conditions (n = 6). G,H) Comet assay of osteosarcoma cells (MNNG, U2OS, K7M2) treated with cisplatin under control or Adi‐CM conditions (n = 40). I) Western blot analysis of apoptosis and DNA damage markers in osteosarcoma cells (MNNG, U2OS, K7M2). J) Western blot analysis of PARP1/ATM pathway proteins in osteosarcoma cells. K) Xenograft tumors formed by MNNG cells mixed with or without adipocytes and treated with cisplatin (0.4 mg/kg) (n = 7). L,M) Volume and weight of xenograft tumors in (K) (n = 7). N) Xenograft tumors formed by K7M2 cells in lean or obese BALB/c mice and treated with cisplatin (0.4 mg kg^−1^) (n = 5). O,P) Volume and weight of xenograft tumors in (N) (n = 5). Q) γH2AX expression in xenograft tumor tissues from (K). R) γH2AX expression in xenograft tumor tissues from (N). The concentration of cisplatin in vitro was 6 µM. *p < 0.05; **p < 0.01; ***p < 0.001; ****p < 0.0001.

Following the identification of cisplatin as a primary chemotherapeutic agent affected by adipocyte‐secreted factors, we sought to elucidate the mechanisms underlying this resistance. First, Adi‐CM reduced apoptosis, as demonstrated by the decreased levels of pro‐apoptotic proteins, including BAX and cleaved caspase‐3 (**Figure** **2**A,B,I).

Cisplatin resistance in tumors often arises through mechanisms such as reduced oxidative stress, modulation of drug transporters, and enhanced DNA repair capacity. We systematically evaluated these pathways to identify the mechanisms underlying adipocyte‐mediated resistance. Adi‐CM treatment did not alter the intracellular reactive oxygen species (ROS) levels or affect the expression of cisplatin transporter proteins, including CTR1 (influx) and P‐gp (efflux) (Figure 2C,D; Figure S4, Supporting Information). However, Adi‐CM significantly inhibited the induction of γH2AX, a well‐established marker of DNA DSBs, in cisplatin‐treated osteosarcoma cells (Figure 2E,F,I). This observation was further corroborated by the comet assay, which revealed a marked reduction in the DNA damage ratio following Adi‐CM treatment (Figure 2G,H). Collectively, these results indicated that adipocyte‐secreted factors mitigate cisplatin‐induced DNA damage.

To investigate whether the reduced DNA damage was attributable to enhanced DNA repair, we assessed the DDR pathway. PARP1, a critical sensor of DNA DSBs, orchestrates DDR by activating key components such as ATM and MRE11. Adi‐CM treatment led to increased expression of PARP1, ATM, and phosphorylated ATM (p‐ATM) in cisplatin‐treated cells both in protein and transcription level (Figure 2J; Figure S5A,B, Supporting Information). Furthermore, downstream effectors of ATM, including p53 and its phosphorylated form (p‐p53), were upregulated, indicating temporary cell cycle arrest to facilitate DNA repair. Rad51, a central mediator of homologous recombination repair (HR), was activated in the presence of Adi‐CM. Notably, pharmacological inhibition of ATM using KU‐55933 effectively reversed cisplatin resistance induced by Adi‐CM (Figure S6, Supporting Information). These findings strongly support the hypothesis that adipocyte‐secreted factors mitigate DNA damage in osteosarcoma cells by enhancing DNA repair.

We used two mouse models to validate the role of adipocytes in promoting cisplatin resistance in vivo. In the first model, a mixture of adipocytes and MNNG cells (1:4) was subcutaneously injected into nude mice. In the second model, we established a diet‐induced obesity (DIO) model that resulted in significant weight gain and increased adipocyte volume (Figure S7A,B, Supporting Information). In both models, under the influence of adipocytes, tumors exhibited accelerated growth, larger volumes, and higher weights after cisplatin treatment compared to the control groups (Figure 2K–P). Consistent with our in vitro findings, γH2AX expression was markedly lower in tumors from both the mixed injection group and the obesity group, even following cisplatin treatment (Figure 2Q,R; Figure S8A–D, Supporting Information). These results confirm that adipocytes confer cisplatin resistance in vivo by mitigating DNA damage.

### 2.3 Adipocyte‐Derived A1BG Drives Cisplatin Resistance in Osteosarcoma via PARP1/ATM‐Mediated DNA Repair

**Figure 3:**
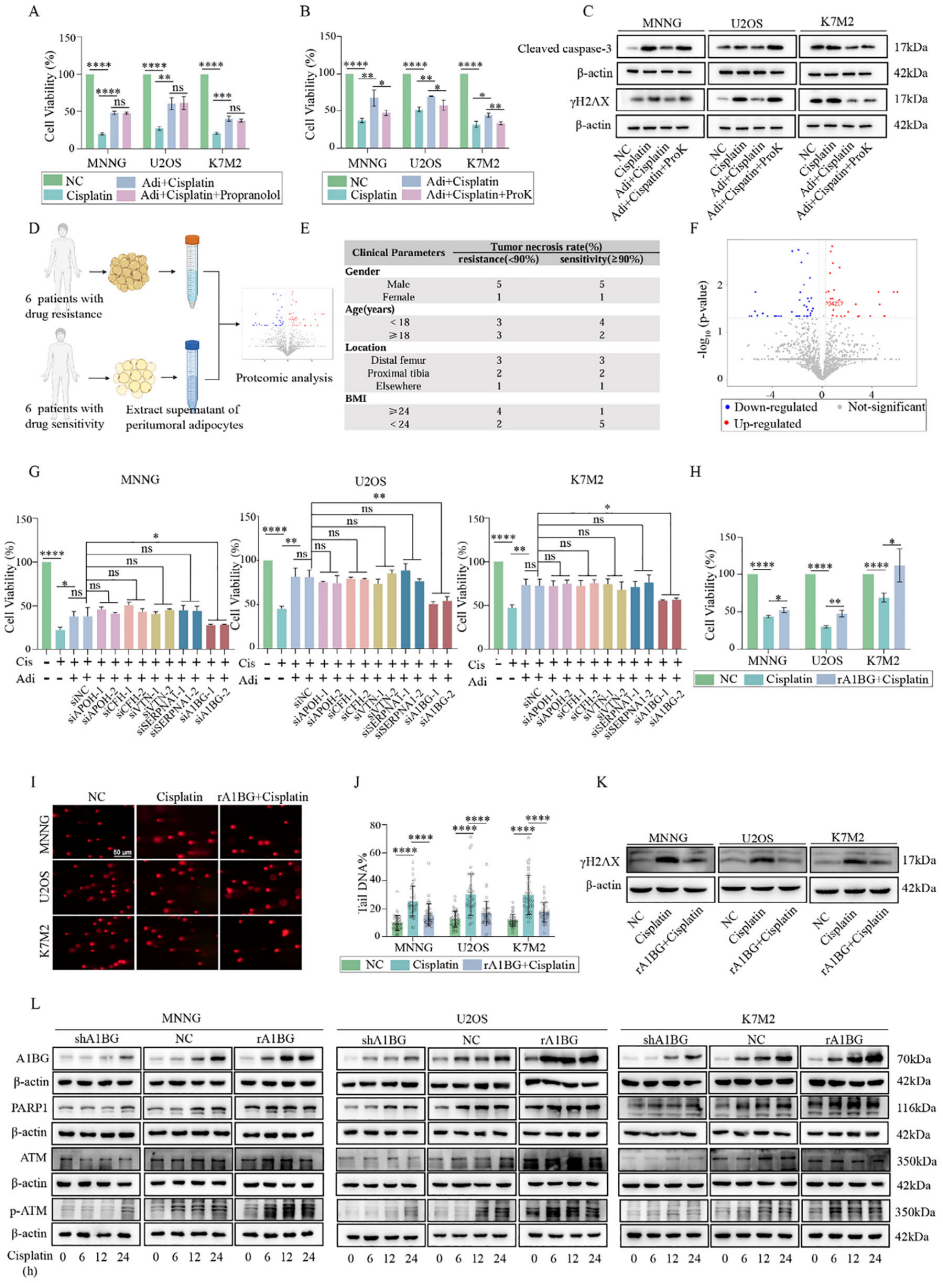
Adipocyte‐Derived A1BG Drives Cisplatin Resistance in Osteosarcoma via PARP1/ATM‐Mediated DNA Repair. A) CCK‐8 assay of osteosarcoma cells treated with cisplatin under control, Adi‐CM, or free fatty acid conditions (n = 3). B) CCK‐8 assay of osteosarcoma cells treated with cisplatin under control, Adi‐CM, or free‐secreted protein conditions (n = 3). C) Western blot analysis of apoptotic and DNA damage markers in osteosarcoma cells treated with cisplatin under control, Adi‐CM, or free‐secreted protein conditions. D) Schematic representation of supernatant collection and proteomic analysis. This figure was created in BioRender. Liang, Y. (2025) https://BioRender.com/e5hptvo. E) Clinical information of 12 patients from whom adipocytes were collected for proteomic analysis. F) Volcano plot depicting differentially expressed proteins identified in proteomic analysis. G) CCK‐8 assay of osteosarcoma cells treated with cisplatin in the presence or absence of Adi‐CM depleted of individual proteins, including APOH, CFH, VTN, SERPINA1, and A1BG (n = 3). H) CCK‐8 assay of osteosarcoma cells treated with cisplatin with or without recombinant A1BG protein (n = 3). I,J) Comet assay of osteosarcoma cells treated with cisplatin with or without recombinant A1BG protein (n = 40). K) γH2AX expression in osteosarcoma cells treated with cisplatin with or without recombinant A1BG protein. L) Western blot analysis of A1BG, PARP1, ATM, and p‐ATM expression at 0, 6, 12, and 24 h following cisplatin treatment in osteosarcoma cells under A1BG‐depleted or recombinant A1BG‐supplemented conditions. The concentration of cisplatin in vitro was 6 µM. *p < 0.05; **p < 0.01; ***p < 0.001; ****p < 0.0001.

To identify adipocyte‐secreted factors responsible for inducing chemoresistance in osteosarcoma cells, we treated osteosarcoma cells with two types of conditioned media: free fatty acid (FFA)‐conditioned medium (collected after propranolol treatment) and protein‐depleted conditioned medium (treated with proteinase K). Notably, FFA‐conditioned medium failed to reverse cisplatin resistance in osteosarcoma cells (**Figure** **3**A). Conversely, proteinase K‐treated conditioned medium lost its ability to confer chemoresistance, as demonstrated by both CCK‐8 assays and the reduced expression of key markers, including γH2AX and cleaved caspase‐3 (Figure 3B,C). These findings strongly suggest that adipocyte‐secreted proteins, rather than free fatty acids, are the primary mediators of cisplatin resistance in osteosarcoma cells.

To identify the specific protein responsible for this effect, we conducted a proteomic analysis of the supernatant from peritumoral adipocytes collected from six chemotherapy‐resistant and six chemotherapy‐sensitive osteosarcoma patients, classified based on a 90% tumor necrosis rate (Figure 3D). The clinical parameters of the patients are summarized in Figure 3E. Differentially expressed proteins were visualized using volcano plots and hierarchical clustering analyses (Figure 3F; Figure S9A, Supporting Information). Additional analyses, including Gene Ontology (GO), Kyoto Encyclopedia of Genes and Genomes (KEGG) pathway enrichment, subcellular localization, and protein‐protein interaction (PPI) network mapping, were performed to further characterize these proteins (Figure S9B–E, Supporting Information).

Among the five most differentially expressed proteins—A1BG, APOH, CFH, VTN, and SERPINA1—we prioritized candidates with a base expression level greater than 0.5 and prior tumor‐related associations reported in the literature.^[^ ^20^ ^]^ Functional assays revealed that conditioned media from A1BG‐depleted adipocytes significantly reduced osteosarcoma cell viability following cisplatin treatment (Figure 3G; Figure S10A–E, Supporting Information), indicating that A1BG is a key driver of adipocyte‐mediated chemoresistance. Moreover, recombinant A1BG treatment significantly enhanced cell viability, reduced DNA damage, and suppressed γH2AX expression in osteosarcoma cells exposed to cisplatin (Figure 3H–K). These findings suggest that A1BG is a critical component of the adipocyte secretome that promotes DNA damage repair and enhances osteosarcoma cell survival.

Previous studies have shown that adipocyte supernatant promotes DNA repair by activating the PARP1/ATM pathway. Therefore, we hypothesized that A1BG facilitates cisplatin resistance by modulating this pathway. To test this, we assessed the expression of A1BG and DNA repair‐related proteins (PARP1, ATM, and p‐ATM) at various time points (0, 6, 12, and 24 h) following cisplatin treatment in osteosarcoma cells under A1BG‐depleted or recombinant A1BG‐supplemented conditions. Western blot analysis revealed that A1BG expression increased over time in cisplatin‐treated osteosarcoma cells. Importantly, A1BG knockdown impaired and delayed activation of the PARP1/ATM pathway, whereas supplementation with recombinant A1BG enhanced its expression and accelerated activation of PARP1, ATM, and p‐ATM (Figure 3L). These findings indicate that A1BG acts as a protective factor by activating the PARP1/ATM pathway and facilitating DNA repair in osteosarcoma cells.

Mechanistically, as DNA damage accumulates during cisplatin treatment, osteosarcoma cells upregulate endogenous A1BG expression to initiate DNA repair. However, this endogenous response is insufficient to rapidly counteract cisplatin‐induced apoptosis. Supplementation with exogenous A1BG significantly accelerates DNA repair, thereby enhancing osteosarcoma cell survival under chemotherapeutic stress. Collectively, our results identify A1BG as a critical adipocyte‐derived protein that mediates cisplatin resistance in osteosarcoma by promoting PARP1/ATM‐dependent DNA repair.

Furthermore, we investigated the specificity and source of A1BG. Our experiments revealed that the expression of A1BG in mature adipocytes was significantly higher than that in bone marrow‐derived mesenchymal stem cells (BMSCs), fibroblasts, and adipose‐derived mesenchymal stem cells (ADSCs) (Figure S11A,B, Supporting Information). Additionally, A1BG expression was progressively upregulated during adipocyte differentiation (Figure S11C,D, Supporting Information). On the other hand, Cancer‐Associated Adipocytes (CAAs) play a crucial role in the tumor microenvironment. To determine whether A1BG expression is influenced by osteosarcoma cells, we conducted experiments and found that the expression of A1BG remained unchanged in CAAs (Figure S12A–D, Supporting Information). These findings demonstrate that A1BG is specifically expressed by adipocytes and that its high expression is an intrinsic characteristic of adipocytes.

### 2.4 Targeting Adipocyte‐Derived A1BG Overcomes Cisplatin Resistance in Osteosarcoma

**Figure 4:**
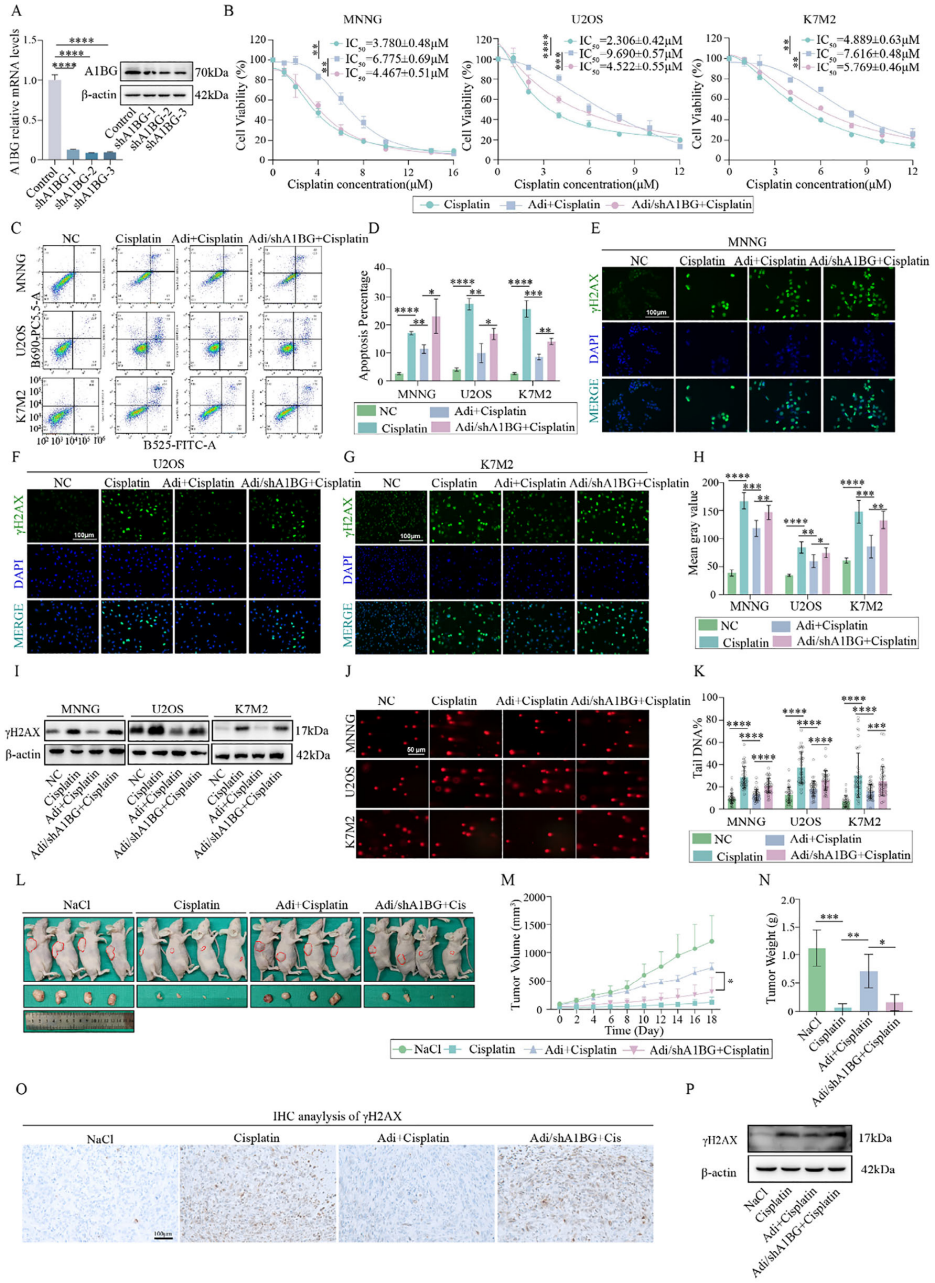
Targeting Adipocyte‐Derived A1BG Overcomes Cisplatin Resistance in Osteosarcoma. A) The qPCR and WB were used to detect the knock down efficiency of A1BG shRNA in 3T3‐L1 cells. B) Osteosarcoma cells (MNNG, U2OS, K7M2) were treated with cisplatin at varying concentrations for 48 h under control, Adi‐CM, and A1BG‐depleted Adi‐CM conditions, and the IC_50_ was calculated (n = 3). C,D) Apoptosis assay of osteosarcoma cells treated with cisplatin under control, Adi‐CM, and A1BG‐depleted Adi‐CM conditions (n = 3). E–H) Immunofluorescence analysis of γH2AX in osteosarcoma cells under the conditions described in (C) (n = 6). I) Western blot analysis of γH2AX in osteosarcoma cells under the conditions described in (C). J,K) Comet assay of osteosarcoma cells under the conditions described in (C) (n = 40). L) Xenograft tumors formed by MNNG cells mixed with or without adipocytes and A1BG‐depleted adipocytes, followed by cisplatin treatment (0.4 mg kg^−1^) (n = 4). M,N) Volume and weight of xenograft tumors from (L) (n = 4). O,P) γH2AX expression in xenograft tumor tissues from (L), analyzed via immunohistochemistry (IHC) and western blot. The concentration of cisplatin in vitro was 6 µM. *p < 0.05; **p < 0.01; ***p < 0.001; ****p < 0.0001.

Building on the established role of A1BG in promoting DNA repair, we investigated the therapeutic potential of targeting adipocyte‐secreted A1BG in osteosarcoma. Stable knockdown of A1BG in 3T3‐L1 adipocytes (shA1BG Adi‐CM) significantly reduced cisplatin resistance in osteosarcoma cell lines (MNNG, U2OS, and K7M2), as evidenced by decreased IC_50_ values compared to control adipocyte‐conditioned medium (Adi‐CM) (**Figure** **4**A,B). This increased sensitivity to cisplatin was further corroborated by increased apoptosis rates in osteosarcoma cells treated with shA1BG Adi‐CM (Figure 4C,D).

Mechanistically, we observed that shA1BG Adi‐CM treatment impaired DNA repair capacity in osteosarcoma cells. Both immunofluorescence and western blot analyses revealed increased γH2AX expression, a marker of DNA DSBs, indicating an elevated accumulation of DNA damage (Figure 4E–I). A comet assay further supported this finding, demonstrating a significant increase in the DNA damage ratio in response to shA1BG Adi‐CM treatment (Figure 4J,K).

To validate these in vitro findings, we established an in vivo model by subcutaneously injecting nude mice with MNNG cells mixed with control or A1BG‐depleted adipocytes. Remarkably, tumors in the A1BG‐depleted group exhibited significantly reduced growth, as evidenced by decreased tumor weight and volume following cisplatin treatment (Figure 4L–N). These findings suggest that blocking A1BG secretion from peritumoral adipocytes effectively enhances cisplatin sensitivity in vivo. Furthermore, immunohistochemistry (IHC) and western blot analyses of tumor tissues confirmed increased γH2AX expression in the A1BG‐depleted group after cisplatin treatment, further indicating enhanced DNA damage in vivo (Figure 4O,P; Figure S13, Supporting Information).

Our findings highlight A1BG as a critical mediator of adipocyte‐induced cisplatin resistance in osteosarcoma. Targeting A1BG secretion from peritumoral adipocytes effectively disrupts DNA damage repair, thereby enhancing cisplatin sensitivity and inhibiting tumor growth in osteosarcoma.

### 2.5 A1BG Interacts with and Stabilizes NAMPT in Osteosarcoma Cells

**Figure 5:**
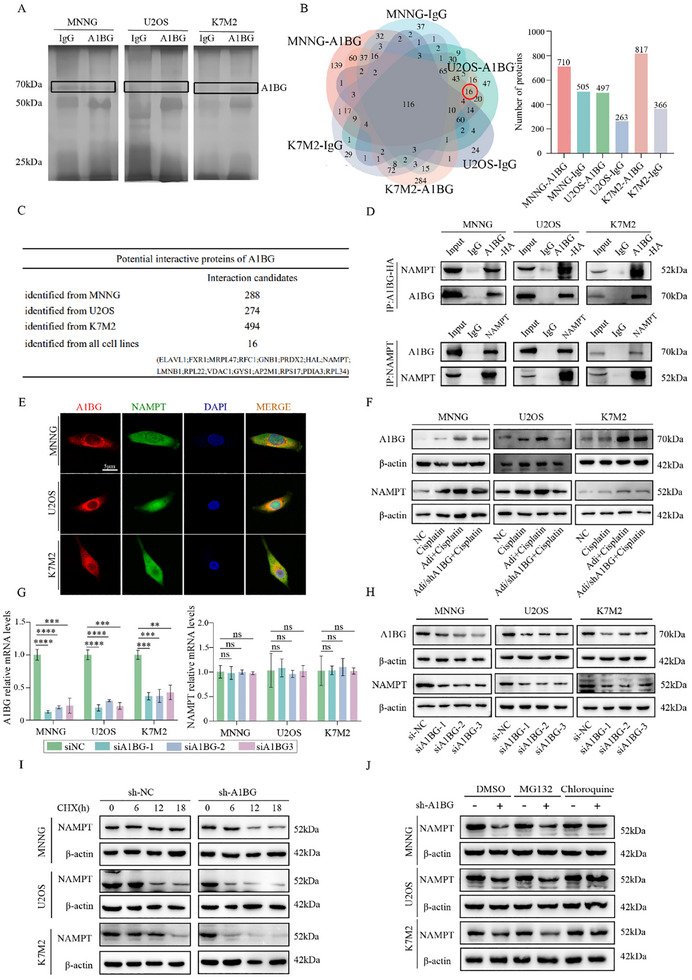
A1BG Interacts with and Stabilizes NAMPT in Osteosarcoma Cells. A) Co‐immunoprecipitation (Co‐IP) assay to determine protein interactions with A1BG. Immunoprecipitated isolated using an anti‐HA antibody were subjected to sodium dodecyl sulfate‐polyacrylamide gel electrophoresis (SDS‐PAGE) followed by silver staining. B) Distribution of protein numbers identified in mass spectrometry (MS) analysis in MNNG‐A1BG, MNNG‐IgG, U2OS‐A1BG, U2OS‐IgG, K7M2‐A1BG, and K7M2‐IgG groups. C) Number of potential A1BG‐interacting proteins identified in MNNG, U2OS, and K7M2 cells. D) Co‐IP using an anti‐HA antibody (upper panel) or anti‐NAMPT antibody (lower panel), followed by immunoblotting with anti‐A1BG and anti‐NAMPT antibodies. IgG served as a negative control. E) Immunofluorescence analysis confirming A1BG and NAMPT colocalization. A1BG was labeled in red, NAMPT in green, and the nucleus in blue. F) A1BG and NAMPT expression levels in osteosarcoma cells treated with cisplatin under control, Adi‐CM, and A1BG‐depleted Adi‐CM conditions. G) NAMPT mRNA levels following A1BG knockdown, showing no significant change. H) NAMPT protein levels following A1BG knockdown, showing a significant decrease. I) Osteosarcoma cells (MNNG, U2OS, K7M2) with or without A1BG knockdown were treated with cycloheximide (50 µg mL^−1^) for 0, 6, 12, and 18 h. Cell lysates were analyzed by western blot. J) The expression of NAMPT with A1BG knockdown or not under the DMSO, MG132 and Chloroquine treatment in MNNG, U2OS and K7M2 cells. **p < 0.01; ***p < 0.001; ****p < 0.0001.

To elucidate the molecular mechanisms underlying A1BG‐mediated DNA repair in osteosarcoma, we sought to identify A1BG‐interacting proteins. Co‐immunoprecipitation (Co‐IP) followed by mass spectrometry (MS) analysis was performed on osteosarcoma cell lines (MNNG, U2OS, and K7M2) overexpressing HA‐tagged A1BG (Figure S14A,B, Supporting Information). This analysis identified 16 proteins that consistently interacted with A1BG in all three cell lines (**Figure** **5**A–C). After excluding nonspecific interactors and conducting a comprehensive literature review focused on DNA repair pathways,^[^ ^21^ ^]^ we selected five candidate proteins—NAMPT, PRDX2, RFC1, LMNB1, and ELAVL1—for further validation.

Co‐IP followed by western blot analysis confirmed a robust interaction between A1BG and NAMPT (Figure 5D), whereas no interactions were detected with the remaining candidates (Figure S15, Supporting Information). Furthermore, immunofluorescence (IF) assays demonstrated colocalization of A1BG and NAMPT in osteosarcoma cells, supporting their direct interaction (Figure 5E). Given the established role of NAMPT in DNA repair, we investigated the functional relationship between A1BG and NAMPT. Consistent with our previous findings, NAMPT protein expression was upregulated in response to Adi‐CM treatment and downregulated upon A1BG depletion (Figure 5F). Interestingly, A1BG knockdown significantly reduced NAMPT protein levels without affecting NAMPT mRNA expression (Figure 5G,H), whereas NAMPT knockdown did not alter A1BG protein levels (Figure S16, Supporting Information). These results suggest that A1BG post‐transcriptionally regulates NAMPT. To confirm this, cycloheximide (CHX) chase assays were performed, revealing that A1BG knockdown significantly reduced the half‐life of NAMPT in osteosarcoma cells (Figure 5I; Figure S17, Supporting Information). Further mechanistic experiments demonstrated that A1BG inhibits lysosome‐mediated degradation of NAMPT (Figure 5J). These findings indicate that A1BG enhances NAMPT stability. Taken together, our results identify NAMPT as a novel A1BG‐interacting protein and demonstrate that A1BG promotes NAMPT protein stability in osteosarcoma cells. These findings suggest that the A1BG‐NAMPT axis plays a critical role in A1BG‐mediated DNA repair.

### 2.6 A1BG‐Activated NAMPT Enhances NAD^+^ Levels and Promotes PARP1‐Mediated ADP Ribosylation and DNA Repair

**Figure 6:**
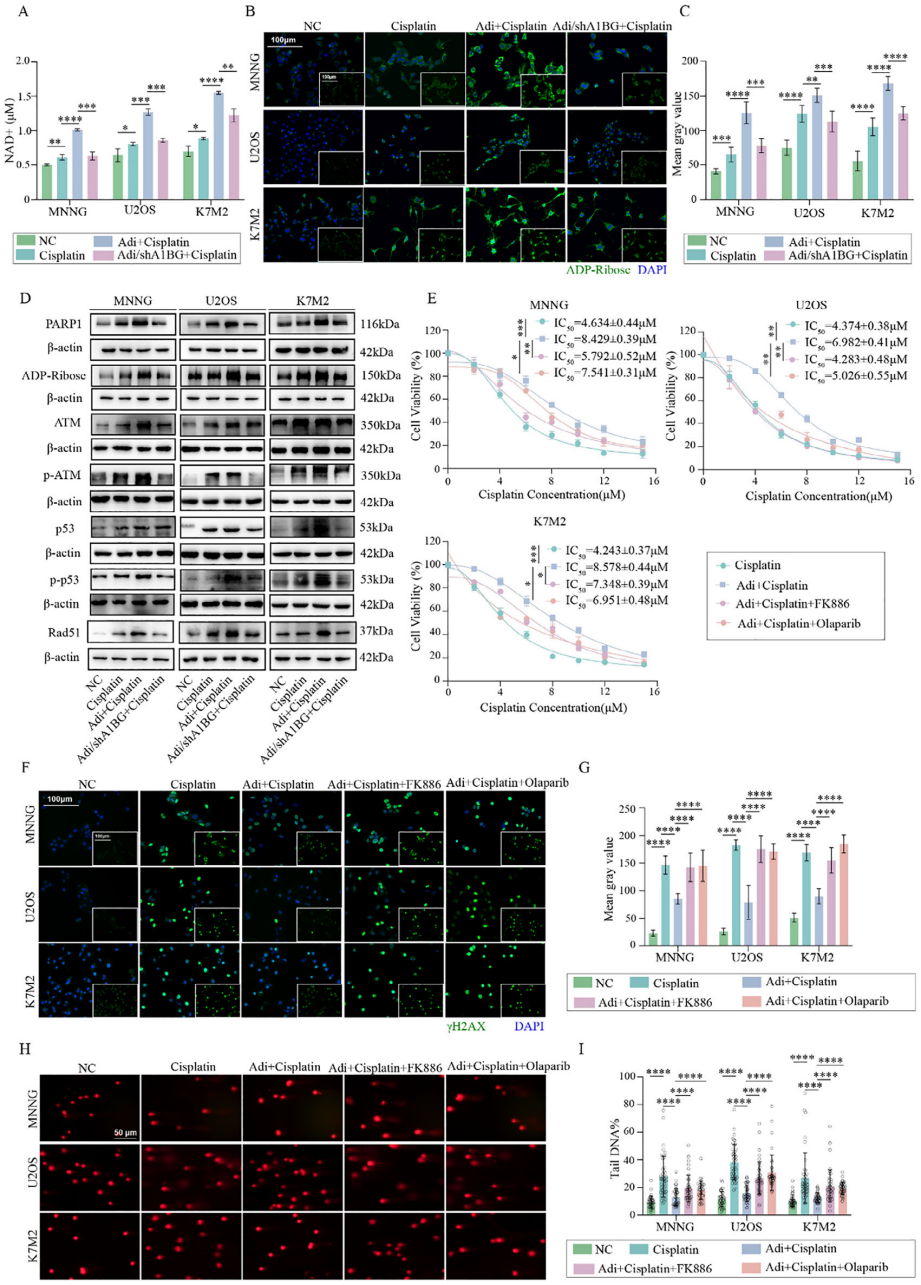
A1BG‐Activated NAMPT Enhances NAD+ Levels and Promotes PARP1‐Mediated ADP‐Ribosylation and DNA Repair. A) NAD⁺ levels in osteosarcoma cells treated with cisplatin under control, Adi‐CM, and A1BG‐depleted Adi‐CM conditions (n = 3). B,C) Immunofluorescence analysis of ADP levels in osteosarcoma cells treated with cisplatin under control, Adi‐CM, and A1BG‐depleted Adi‐CM conditions (n = 6). D) Western blot analysis of PARP1/ATM pathway proteins in osteosarcoma cells. E) IC_50_ values of cisplatin in osteosarcoma cells treated with or without Adi‐CM, FK886 (5 nM), and Olaparib (10 µM) (n = 3). F,G) Immunofluorescence analysis of γH2AX in osteosarcoma cells under the conditions described in (E) (n = 6). H,I) Comet assay of osteosarcoma cells under the conditions described in (E) (n = 40). The concentration of drug in vitro: cisplatin = 6 µM; FK886 = 5 nM; Olaparib = 10 µM. *p < 0.05; **p < 0.01; ***p < 0.001; ****p < 0.0001.

Given the established roles of NAMPT in NAD⁺ biosynthesis and PARP1‐mediated ADP ribosylation, both of which are critical for efficient DNA repair, we investigated whether A1BG enhances DNA repair through this pathway. We hypothesized that A1BG stimulates NAMPT activity, leading to increased NAD⁺ levels and subsequent activation of PARP1, thereby promoting DNA repair and contributing to cisplatin resistance in osteosarcoma. Supporting this hypothesis, we observed a significant increase in NAD⁺ levels in osteosarcoma cells treated with Adi‐CM, which was abrogated by A1BG depletion following cisplatin treatment (**Figure** **6**A).

Furthermore, Adi‐CM treatment enhanced ADP ribosylation, as evidenced by both immunofluorescence and western blot assays, whereas A1BG depletion reversed this effect (Figure 6B–D). Consistent with these observations, Adi‐CM treatment increased PARP1 protein levels, which were abrogated by A1BG depletion (Figure 6D). Similarly, A1BG depletion attenuated activation of the PARP1/ATM pathway, further supporting its role in DDR signaling (Figure 6D).

To confirm the functional relevance of the NAMPT/PARP1 pathway in A1BG‐mediated chemoresistance, we evaluated the effects of the NAMPT and PARP1 inhibitors FK886 and Olaparib, respectively. As expected, Adi‐CM treatment increased the IC_50_ of cisplatin in osteosarcoma cells. However, co‐treatment with either FK886 or Olaparib significantly reduced IC_50_ values, indicating re‐sensitization to cisplatin (Figure 6E). Furthermore, both inhibitors markedly decreased ADP ribosylation, increased γH2AX fluorescence intensity and DNA damage, and promoted cellular apoptosis in Adi‐CM and cisplatin‐treated cells. These results confirm the ability of FK866 and Olaparib to block adipocyte‐induced DNA repair and cisplatin resistance in osteosarcoma cells (Figure 6F–I; Figure S18A–D, Supporting Information). These findings demonstrate that targeting NAMPT and PARP1 effectively reverses drug resistance and suppresses adipocyte‐induced DNA repair in osteosarcoma cells.

Additionally, to further investigate the roles of FK866 and Olaparib in the osteosarcoma microenvironment, we established a co‐culture model of adipocytes and osteosarcoma cells, as well as a peritumoral adipocyte‐conditioned medium treatment model. Experimental results showed that both models enhanced cisplatin resistance in osteosarcoma cells. However, treatment with FK866 or Olaparib effectively inhibited this phenomenon (Figure S19A,B, Supporting Information). These findings indicate that CAAs induce cisplatin resistance through the A1BG/NAMPT/PARP1 axis and that inhibiting NAMPT and PARP1 can enhance cisplatin sensitivity in osteosarcoma cells.

### 2.7 Targeting NAMPT and PARP1 Reduces Cisplatin Resistance Induced by Adipocytes in Osteosarcoma

**Figure 7:**
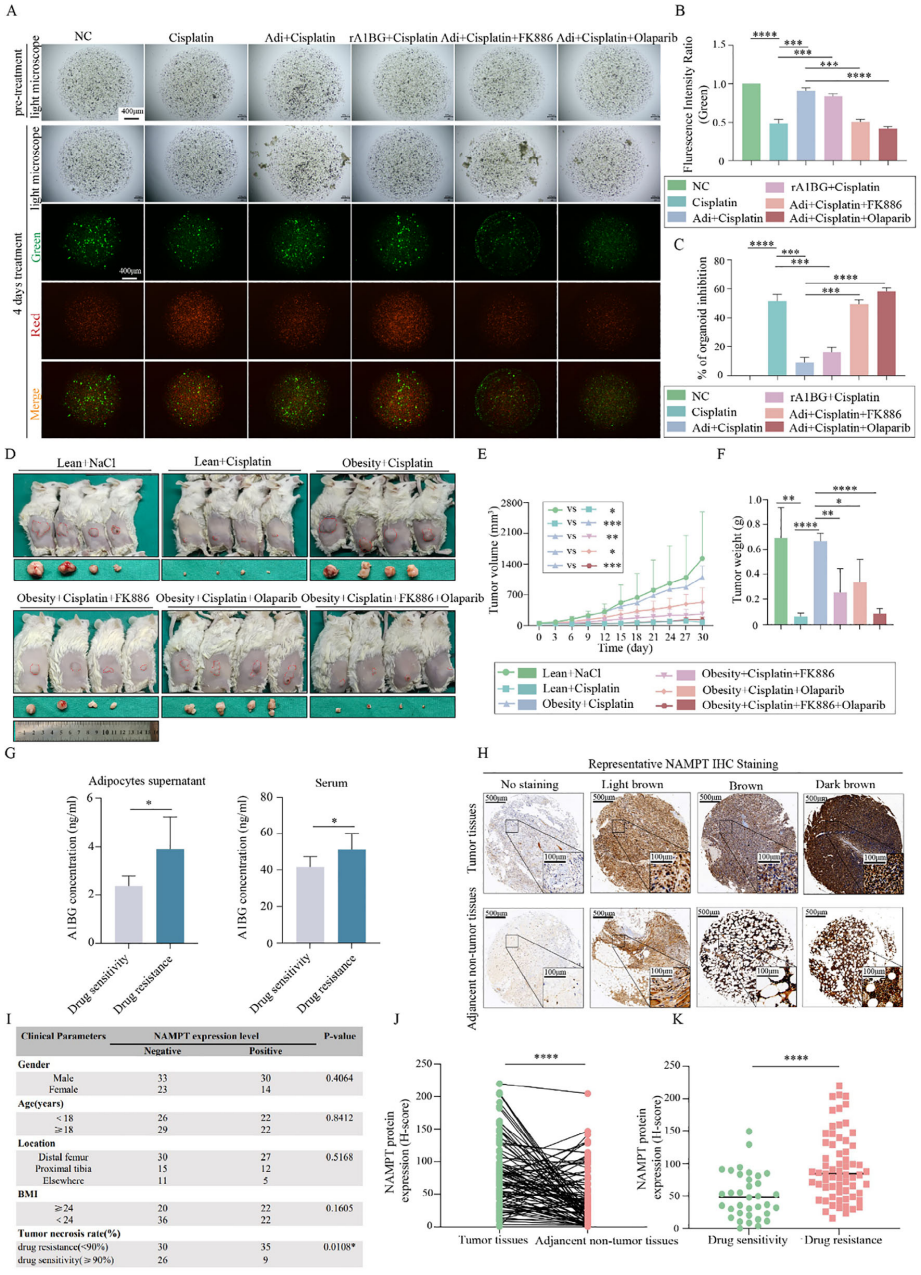
Targeting NAMPT and PARP1 Reduces Cisplatin Resistance Induced by Adipocytes in Osteosarcoma. A) Assessment of cisplatin resistance induced by Adi‐CM and A1BG, and evaluation of the therapeutic efficacy of FK886 and Olaparib in an osteosarcoma organoid model (n = 3). B) Cell viability analysis in the osteosarcoma organoid model using live/dead staining. C) Quantification of organoid inhibition rate from (A). D) Evaluation of cisplatin treatment in combination with FK886 and Olaparib in a xenograft tumor model using obese mice (n = 4). E,F) Tumor volume and weight measurements from xenograft tumors in (D). G) ELISA analysis of A1BG levels in peritumoral adipocyte supernatants and serum samples (n = 6). H) Tissue microarray (TMA) analysis of NAMPT expression in osteosarcoma tissues, with a representative IHC‐stained image. I) Correlation analysis between NAMPT expression levels and clinical variables in 100 patients with osteosarcoma. J) Immunohistochemistry (IHC) analysis quantifying NAMPT expression in osteosarcoma tissues and matched adjacent normal tissues. K) IHC analysis comparing NAMPT expression levels in chemoresistant and chemosensitive osteosarcoma tumors. The concentration of drug in vitro: cisplatin = 6 µM; FK886 = 5 nM; Olaparib = 10 µM. *p < 0.05; **p < 0.01; ***p < 0.001; ****p < 0.0001.

To validate our findings in a clinically relevant setting, we first assessed the effects of NAMPT and PARP1 inhibition in patient‐derived osteosarcoma organoids. Consistent with our in vitro results, cisplatin effectively inhibited organoid growth, whereas treatment with Adi‐CM or recombinant A1BG significantly increased resistance to cisplatin. Importantly, this resistance was abrogated by co‐treatment with either FK886 or Olaparib, confirming the clinical relevance of targeting this pathway (**Figure** **7**A–C).

To further evaluate the therapeutic potential of NAMPT and PARP1 inhibition in vivo, we employed a DIO model of osteosarcoma. The body weight of mice in each group is shown in Figure S20 (Supporting Information). As expected, tumors in obese mice exhibited increased resistance to cisplatin compared to those in lean mice. However, combined treatment with cisplatin and FK886, Olaparib, or both significantly reduced tumor volume and weight compared to cisplatin alone (Figure 7D–F). Furthermore, combination therapy significantly increased γH2AX expression, indicating enhanced DNA damage (Figure S21A,B, Supporting Information).

To explore the clinical relevance of the A1BG‐NAMPT axis in patients with osteosarcoma, we analyzed A1BG levels in peritumoral adipocyte supernatants and serum samples from patients stratified by their response to chemotherapy. ELISA revealed significantly elevated A1BG levels in both supernatant and serum samples from chemoresistant patients compared to those from chemosensitive patients (Figure 7G). Furthermore, immunohistochemical (IHC) analysis of a tissue microarray (TMA) comprising 100 paired osteosarcoma and normal tissue samples revealed that NAMPT expression was significantly higher in tumor tissues than in matched normal tissues (Figure 7J) and negatively correlated with the tumor necrosis rate (Figure 7I). Importantly, NAMPT expression was significantly higher in chemoresistant tumors than in chemosensitive tumors (Figure 7K). Representative IHC staining results are shown in Figure 7H. On the other hand, we investigated the clinical relationship between obesity and NAMPT expression. A semiquantitative IHC analysis (H‐score) revealed that NAMPT expression was higher in overweight patients (Figure S22, Supporting Information), indicating a positive correlation between NAMPT expression and obesity.

Taken together, these findings demonstrate that adipocyte‐derived A1BG promotes cisplatin resistance in osteosarcoma by activating the NAMPT/PARP1 pathway. Targeting this pathway using NAMPT or PARP1 inhibitors represents a promising therapeutic strategy to overcome chemoresistance in osteosarcoma.

## 3 Discussion

Osteosarcoma, a prevalent malignant bone tumor, remains a persistent clinical challenge due to its poor prognosis, with minimal improvement in overall survival over the past four decades. This stagnation is largely attributed to the development of chemoresistance, which remains a major obstacle to successful treatment.^[^ ^22^ ^]^ Although tumor‐intrinsic factors driving chemoresistance have been extensively studied, the contribution of systemic factors, particularly obesity, remains underexplored. The impact of obesity on tumors is primarily mediated by the accumulation of peritumoral adipocytes, which create a tumor‐promoting microenvironment that induces chemoresistance through secreted factors such as adipokines,^[^ ^6b^ ^]^ exosomes,^[^ ^23^ ^]^ and proteins.^[^ ^24^ ^]^ However, the specific effects of adipocytes in osteosarcoma therapy remain unclear. This study reveals a novel mechanism by which obesity, through the abundant peritumoral adipocytes characteristic of tumors in obese individuals, promotes cisplatin resistance in osteosarcoma, highlighting the A1BG/NAMPT/PARP1 axis as a promising therapeutic target.

Our investigation was prompted by the clinical observation of a strong correlation between BMI and increased chemoresistance in osteosarcoma patients. This finding led us to explore the effect of adipocytes, key components of the obese tumor microenvironment, on the response of osteosarcoma cells to chemotherapy. We demonstrated that Adi‐CM selectively protects osteosarcoma cells from the cytotoxic effects of platinum‐based drugs, including cisplatin, carboplatin, and oxaliplatin, suggesting a targeted mechanism affecting this class of chemotherapeutics.

To elucidate this mechanism, we first confirmed that Adi‐CM reduced both in vitro apoptosis and in vivo cisplatin sensitivity, indicating that enhanced DNA repair is a potential driver of resistance. Mechanistically, we found that adipocytes enhance DNA repair by upregulating PARP1, a key DDR protein. This finding aligns with previous reports demonstrating increased expression of DNA repair proteins such as XRCC1 and DNA‐PK in cisplatin‐resistant cells.^[^ ^25^ ^]^ While adipocytes have been implicated in radiotherapy resistance through SERPINE1‐mediated DNA repair,^[^ ^7a^ ^]^ our study is the first to directly link adipocytes to the attenuation of cisplatin‐induced DNA damage. Furthermore, our results indicate that adipocytes activate the PARP1 pathway, which is essential for DNA repair. PARP1 acts as a “sensor” that recruits ATM (“transducer”) to initiate early DDR signaling.^[^ ^26^ ^]^ Inhibition of PARP1 negatively regulates ATM kinase activity in response to DSBs.^[^ ^27^ ^]^

Having established the role of adipocytes in promoting DNA repair and cisplatin resistance, we sought to identify the specific factors mediating this effect. Through a series of exclusion experiments and proteomic analyses, we identified adipocyte‐secreted A1BG as a critical driver. A1BG, a secreted glycoprotein often overexpressed in cancer,^[^ ^17^, ^28^ ^]^ has been linked to poor outcomes in cholangiocarcinoma;^[^ ^29^ ^]^ however, its role in osteosarcoma chemoresistance remains unknown. Our study is the first to demonstrate that exogenous A1BG, supplied by adipocytes, significantly enhances DNA repair and drives cisplatin resistance in osteosarcoma. Importantly, our findings suggest that A1BG acts as a self‐protective factor in osteosarcoma cells. Although intrinsic A1BG signaling is insufficient for rapid DNA repair following cisplatin treatment, exogenous A1BG supplied by adipocytes contributes to chemoresistance. Blocking A1BG secretion effectively enhances DNA damage and mitigates cisplatin resistance, highlighting A1BG as a valuable therapeutic target in patients with obesity and drug resistance.

By delving deeper into this mechanism, we uncovered a novel interaction between A1BG and NAMPT, the rate‐limiting enzyme in NAD⁺ biosynthesis. A1BG directly interacted with and stabilized NAMPT by inhibiting the lysosome‐mediated protein degradation, thereby increasing intracellular NAD⁺ levels. This finding is significant because NAMPT is crucial for maintaining NAD⁺ pools, which are essential for various cellular processes, including DNA repair.^[^ ^30^ ^]^ NAMPT overexpression is frequently observed in cancer and is often associated with chemoresistance.^[^ ^31^ ^]^ In addition to regulating NAD⁺ levels, studies have reported that NAMPT modulates cancer stem cell properties and chemoresistance through SIRT1 and PARP1.^[^ ^31c^ ^]^ Our findings suggest that by stabilizing NAMPT, adipocyte‐derived A1BG increases NAD⁺ availability, thereby fueling PARP1‐mediated ADP ribosylation and ultimately enhancing DNA repair, leading to cisplatin resistance.

Cancer‐associated adipocytes (CAAs) play a crucial role in the tumor microenvironment. Our study reveals that A1BG overexpression is a universal characteristic of adipocytes, and CAAs similarly induce cisplatin resistance in osteosarcoma through the A1BG/NAMPT/PARP1 axis. Targeting NAMPT and PARP1 remains a viable therapeutic strategy in both CAAs and normal adipocytes. These findings significantly enhance the clinical potential of NAMPT and PARP1 inhibitors to reverse cisplatin resistance in obese patients with osteosarcoma.

These findings have significant therapeutic implications for obese patients with osteosarcoma, who are at an increased risk of chemoresistance. Our study provides a strong rationale for targeting the A1BG/NAMPT/PARP1 axis to overcome this challenge. This can be achieved by developing A1BG inhibitors or employing existing NAMPT and PARP1 inhibitors in combination with standard chemotherapy. Our in vitro, in vivo, and organoid models demonstrated the efficacy of the NAMPT inhibitor FK886 and the PARP1 inhibitor Olaparib in mitigating cisplatin resistance, supporting the potential of this therapeutic strategy. Furthermore, our data suggest that A1BG could serve as both a therapeutic target and a potential serum biomarker for predicting cisplatin resistance in osteosarcoma, paving the way for personalized treatment approaches. While PARP1 has emerged as a promising target for overcoming chemoresistance in osteosarcoma, a clinical study demonstrated encouraging anti‐tumor activity when combined with trabectedin in 50 patients,^[^ ^32^ ^]^ its benefits are not universal due to complex molecular mechanisms.^[^ ^33^ ^]^ Our study provides a rationale for the use of PARP1 inhibitors in obese patients with osteosarcoma. Additionally, our tissue microarray data suggest that NAMPT could serve as a postoperative pathological indicator for predicting drug resistance in these patients.

## 4 Conclusion

In conclusion, our study revealed a novel mechanism by which obesity promotes cisplatin resistance in osteosarcoma through the presence of abundant peritumoral adipocytes. We demonstrated that adipocyte‐secreted A1BG enhances DNA repair by stabilizing NAMPT, leading to increased NAD⁺ levels and activation of the PARP1/ATM pathway. These findings provide a strong rationale for targeting the A1BG/NAMPT/PARP1 axis to improve clinical outcomes in obese patients with osteosarcoma.

## 5 Experimental Section

### Cell Culture and Reagents

Human osteosarcoma cell lines U2OS and MNNG/HOS, the mouse osteosarcoma cell line K7M2, and the mouse preadipocyte cell line 3T3‐L1 were obtained from the Shanghai Institute for Biological Sciences. All cell lines were cultured according to the American Type Culture Collection (ATCC) protocol and tested negative for Mycoplasma contamination.

Cisplatin (S1166), doxorubicin (E2516), methotrexate (S1210), ifosfamide (S1302), KU‐55933 (S1092), daporinad (FK866) (S2799), and Olaparib (S1060) were obtained from Selleck.

### Cell Viability Assay and IC_50_ Calculation

Osteosarcoma cells were seeded in 96‐well plates at a density of 5 × 10^3^ cells well^−1^ in 200 µL of culture medium and incubated for 24 h. Following incubation, cells were treated with various reagents according to the specific experimental design. After 48 h of treatment, cell viability was assessed using the Cell Counting Kit‐8 assay (CCK‐8, Share‐bio, SB‐CCK8). Briefly, 10 µL of CCK‐8 working solution was added to each well, followed by a 2 h incubation. Absorbance was measured at 450 nm using a microplate reader. Cell viability was calculated using the formula:

(1) $$\frac{A_{s}-A_{b}}{A_{c}-A_{b}}\times 100\%$$

where *As* is the absorbance of the sample, *Ac* is the absorbance of the negative control sample, and *A_b_* is the absorbance of the blank sample

As to the IC_50_ value, the dose‐response curve was plotted first and the IC_50_ was calculated by nonlinear regression analysis in GraphPad Prism 9.

### Animal Experiments

All animal experiments were conducted in accordance with guidelines approved by the Shanghai Sixth's Hospital Animal Care And Use Committee (No:2024‐0433).

### Subcutaneous Xenograft Studies

Four‐week‐old female BALB/c nude mice were used for subcutaneous xenograft studies. MNNG cells (1 × 10^6^) were mixed with either phosphate‐buffered saline (PBS), mature adipocytes, or 3T3‐L1 cells (0.25 × 10^6^) at a 4:1 ratio. The cell mixture was implanted subcutaneously into the right flank of each mouse. Mice received intraperitoneal injections of either cisplatin or PBS, and tumor size was measured every two days. After two weeks, mice were euthanized, and tumor tissues were collected for further analysis. Tumor volume was calculated using the formula:

(2) $$\left(\mathrm{length}\;\times \;\mathrm{widt}h^{2}\right)\times 0.52$$

### Diet‐Induced Obesity (DIO) Mouse Model

Four‐week‐old female BALB/c mice were randomly assigned to two groups and fed either a control diet (CD, 10% kcal from fat; XTCON50J, Jiangsu Xietong Pharmaceutical Bioengineering Company) or a high‐fat diet (HFD, 60% kcal from fat; XTHF60). Once established on their respective diets, mice were injected with 8 × 10^6^ K7M2 cells into the inguinal white fat pad. Mice in each diet group were further randomized into treatment groups. When tumors reached ≈50 mm^3^, mice received intraperitoneal injections of cisplatin (0.4 mg kg^−1^, 100 µL), FK886 (10 mg kg^−1^, 100 µL), or Olaparib (50 mg kg^−1^, 100 µL) every two days. Body weight, food intake, and tumor volume were monitored every three days until the experimental endpoint. Mice were euthanized when the largest tumor reached ≈1000 mm^3^. Tumors were excised and weighed following euthanasia.

### Adipocyte Differentiation and Collection of Conditioned Medium

For differentiation, 3T3‐L1 cells were seeded at a density of 30 000 cells cm^2^ in DMEM and incubated for 48 h until reaching 100% confluence. Differentiation was induced using DMEM supplemented with 10% fetal bovine serum (FBS), 1% penicillin‐streptomycin, 1% L‐glutamine, 1 µg mL^−1^ insulin (10 mg mL^−1^ insulin solution, Procell, PB180432), 0.25 µM dexamethasone (DEX) (Selleck, S1322), 2 µM rosiglitazone (Selleck, S2556), and 0.5 mM isobutyl‐methyl‐xanthine (IBMX) (Selleck, S5836).After 48 h, the differentiation medium was replaced with maintenance medium (DMEM containing 4.5 g L^−1^ glucose, 10% FBS, 1% penicillin‐streptomycin, 1% L‐glutamine, and 1 µg mL^−1^ insulin). Cells were re‐supplemented with maintenance medium every 48 h until day 15. Following lipid accumulation, cells were incubated in serum‐free medium for 24 h. The conditioned medium was then collected and stored at −20 °C.

### Transfection

Cells (2.0 × 10⁴) were seeded in six‐well plates. The siRNA and lentiviral vectors were transfected according to the manufacturer's instructions. The siRNA and lentiviral vectors were purchased from Genomeditech (Shanghai, China). Transfections were performed using the Lipofectamine 3000 Transfection Kit (Invitrogen). Following lentiviral vector transfection, puromycin (Beyotime, ST551) was used to generate stable cell lines.

### Flow Cytometry Analysis of Apoptosis

Osteosarcoma cells were seeded in six‐well plates (3 × 10^5^ cells well^−1^) and treated with cisplatin or other reagents for 48 h. After treatment, cells were collected, washed, and stained using the Annexin V‐FITC Apoptosis Detection Kit (Beyotime, C1062S) according to the manufacturer's instructions. Apoptosis was analyzed using a flow cytometer (BD Biosciences, Franklin Lakes, NJ, USA).

### Immunofluorescence Staining and Imaging

Osteosarcoma cells were seeded in 24‐well plates (3 × 10^4^ cells well^−1^) and treated with cisplatin or other reagents. γH2AX detection was performed according to the manufacturer's guidelines (DNA Damage Assay Kit by γ‐H2AX Immunofluorescence, Beyotime, C2035S). Images were captured using a fluorescence microscope (DMi8 Inverted Microscope, Leica). The mean fluorescence intensity of six images per group was analyzed using ImageJ Software (National Institutes of Health (NIH), Bethesda, Maryland, United States).

For A1BG, NAMPT, and ADP ribose detection, cells were fixed with 4% paraformaldehyde and blocked using QuickBlock Blocking Buffer (Beyotime, P0220). The following primary and secondary antibodies were used:

- Primary Antibodies:
  - A1BG Polyclonal Antibody (1:100, Proteintech, 14181‐1‐AP)
  - NAMPT Monoclonal Antibody (1:100, Proteintech, 66385‐1‐Ig)
  - Poly/Mono‐ADP Ribose Rabbit mAb (1:3200, CST, 89190S)
- Secondary Antibodies:
  - Goat Anti‐Rabbit IgG H&L AF488 (1:500, Zenbio, 550 037)
  - Goat Anti‐Mouse IgG H&L AF488 (1:500, Zenbio, 550 036)
  - Goat Anti‐Rabbit IgG H&L AF594 (1:500, Sharebio, SB‐AB0151)
- Nuclear Staining:
  - DAPI Staining Solution (Beyotime, C1005)

### Comet Assay

Osteosarcoma cells were seeded in six‐well plates (3 × 10^5^ cells well^−1^) and treated with cisplatin or other reagents. The assay was performed according to the manufacturer's guidelines (Comet Assay Kit, Beyotime, C2041 M). Briefly, cells were harvested by trypsinization, embedded in 0.8% SeaPlaque low‐melting‐point agarose on two‐well comet slides, and lysed overnight at 4 °C in lysis buffer (2.5 M NaCl, 100 mM EDTA, and 10 mM Tris, pH 10) supplemented with 10% DMSO and 1% Triton X‐100. For the alkaline comet assay, slides were washed after lysis and incubated for 40 min in denaturation buffer (300 mM NaOH, 1 mM EDTA), followed by electrophoresis for 20 min at 18 V and 300 mA. After electrophoresis, slides were washed with PBS, fixed in ice‐cold ethanol for 10 min, and dried at 37 °C. DNA was stained with SYBR Gold for 10 min, washed with PBS, and dried at 37 °C. Samples were imaged using a fluorescence microscope (DMi8 Inverted Microscope, Leica) and analyzed using CASP software.

### Western Blot Assay and COIP Assay

Cells or tissues were lysed using a Protein Extraction Reagent Kit (Thermo Fisher Scientific) according to the manufacturer's instructions. Protein concentrations were determined using the bicinchoninic acid (BCA) assay. Following gel electrophoresis, proteins were transferred onto polyvinylidene difluoride (PVDF) membranes. Membranes were blocked with 5% skimmed milk and probed with specific antibodies.

For co‐IP, lysates of 1 × 10^7^ MNNG, U2OS and K7M2 cells were immunoprecipitated with IP buffer containing IP antibody and beads (Protein A/G beads, Share‐bio, SB‐PR001), and protein–protein complexes were later subjected to Western blot. Here, IgG was used as a negative control.

The following antibodies were used:

- IP Antibodies:
  - NAMPT Monoclonal Antibody (Proteintech, 66385‐1‐Ig)
  - HA Tag Recombinant antibody (Proteintech, 81290‐1‐RR)
- Primary Antibodies:
  - FABP4 Rabbit mAb (1:1000, ABclonal, A11481)
  - Cleaved Caspase‐3 Rabbit mAb (1:1000, CST, 9664T)
  - Bax Rabbit mAb (1:1000, ABclonal, A19684)
  - Phospho‐Histone H2AX (Ser139) Rabbit mAb (1:1000, ABclonal, AP0687)
  - ATM Rabbit mAb (1:1000, CST, 2873T)
  - Phospho‐ATM (Ser1981) Rabbit mAb (1:1000, ABclonal, AP1030)
  - p53 Rabbit mAb (1:1000, CST, 2527T)
  - Phospho‐p53 (Ser15) Rabbit mAb (1:1000, CST, 9284)
  - CTR1/SLC31A1 Rabbit mAb (1:1000, ABclonal, A0773)
  - P‐Glycoprotein Rabbit mAb (1:1000, ABclonal, A19093)
  - A1BG Polyclonal Antibody (1:1000, Proteintech, 14181‐1‐AP)
  - NAMPT Monoclonal Antibody (1:1000, Proteintech, 66385‐1‐Ig)
  - PARP1 Polyclonal Antibody (1:1000, Proteintech, 13371‐1‐AP)
  - PRDX2 Polyclonal Antibody (1:1000, Proteintech, 10545‐2‐AP)
  - RFC1 Polyclonal Antibody (1:1000, Proteintech, 19159‐1‐AP)
  - LMNB1 Polyclonal Antibody (1:1000, Proteintech, 12987‐1‐AP)
  - ELVAL1 Polyclonal Antibody (1:1000, Proteintech, 11910‐1‐AP)
- Secondary Antibodies:
  - HRP‐Goat Anti‐Rabbit Antibody (1:10000, Proteintech, RGAR001)
  - HRP‐Goat Anti‐Mouse Antibody (1:10000, Proteintech, RGAM001)
- Loading Control:
  - β‐Actin Antibody (HRP‐conjugated, Sharebio, SB‐AB2001)
  - Rabbit IgG Isotype Control (Zenbio, A00002)

### CHX Chase Assay

For the CHX chase assay, MNNG, U2OS and K7M2 cells were treated with CHX (50 µg ml^−1^) and harvested at the indicated timepoints. Treated cells were lysed, and the lysates were analyzed by Western Blotting

### The Collection of the Supernatant of Human Mature Adipocytes, and the Proteomic Sequencing

For the isolation and collection of human mature adipocytes, adipose tissue was harvested from patients undergoing surgery. The tissue was minced into ≈1 cm pieces and digested with collagenase type I (Solarbio, C8140) for 2 h in an isothermal shaker water circulator at 37 °C. The adipocyte suspension was then mixed with an equal volume of DMEM and centrifuged at 400 × *g* for 10 min at 4 °C. After centrifugation, mature adipocytes were separated from the medium, collected, washed with PBS and incubated in a 6 cm dish with 5 mL of serum‐free medium. After 24 h, the supernatant was collected and stored at −80 °C. Once all samples were collected, they were mixed with 1 mL of lysis buffer, ground at low temperature for 5 min, and sonicated in an ice bath for 5 min. After incubation with rotation at −4 °C for 10 min, the mixture was centrifuged at 12 000 × *g* for 15 min at 4 °C, and the supernatant was collected. Protein concentration was measured using the bicinchoninic acid (BCA) assay. For proteomic analysis, 100 µg of protein from each sample was subjected to acetone precipitation. The precipitate was resuspended in 100 µL of protein redissolution solution and co‐incubated with 5 mM dithiothreitol (DTT) for 10 min at 55 °C, followed by incubation with 10 mM iodoacetamide (IAA) for 15 min. Protein digestion and SDS clean‐up were performed to obtain peptides, which were further desalted for subsequent analysis. Nano‐LC‐MS/MS analysis of peptides was performed by Xunyin Biomedical Technology (Shanghai, China).

### Clinical Samples and IHC

Twelve peritumoral adipose tissue samples were collected from patients undergoing surgical resection at Shanghai No. 6 Hospital in 2024 and were used for proteomic analysis (the Shanghai Sixth's Hospital Independent Ethics Committee, 2024‐YS‐133). Additionally, 100 patients with confirmed osteosarcoma who underwent surgical resection at Shanghai No. 6 Hospital between 2014 and 2017, and for whom postoperative pathological tissues were available, were selected for IHC analysis. A tissue microarray (TMA) containing 100 osteosarcoma tissues and matched normal tissues was prepared by the Department of Orthopedics. All patients provided informed consent for the analysis of their tumor samples, following a protocol approved by the Review Committee of Shanghai No. 6 Hospital. Relevant clinical information was collected.

For IHC staining, tissue slides were dewaxed in xylene and rehydrated using graded ethanol (100%, 95%, and 75%). Antigen retrieval was performed by boiling the slides in EDTA solution for 20 min, followed by cooling for 10 min. After blocking, sections were incubated with primary antibodies for 1 h at 37 °C, followed by incubation with secondary antibodies (Invitrogen, USA) for 30 min. Diaminobenzidine (DAB) was used as the chromogen for 5 min, followed by hematoxylin staining for 2 min. Slides were then rinsed with PBS, counterstained with lithium carbonate, and mounted with coverslips and glycerin.

The percentage of positively stained cells and the staining intensity were used as evaluation criteria. The percentage of positively stained cells was scored as follows:

- 0: <5%
- 1: 5–25%
- 2: 25–50%
- 3: 50–75%
- 4: >75%

The staining intensity was scored as follows:

- 0: No staining
- 1: Light brown
- 2: Brown
- 3: Dark brown

The final IHC scores were obtained using both traditional scoring and the H‐score method:

- In the traditional scoring method, IHC scores were calculated as the product of intensity (0–3) and the percentage of positively stained cells (0–4), yielding a range of 0 to 12. High expression (positive expression) was defined as an IHC score ≥8, while low expression (negative expression) was defined as an IHC score <8.
- The H‐score was calculated using the formula:
(3) $$\left(\%\mathrm{light}\;\mathrm{brown}\times 1\right)+\left(\%\mathrm{brown}\times 2\right)+\left(\%\mathrm{dark}\;\mathrm{brown}\times 3\right)$$

yielding a range from 0 to 300.

### Osteosarcoma Organoid Culture and Live‐Dead Staining

Patient‐derived osteosarcoma organoids were cultured following an organoid culture protocol using media provided by OneTar Biomedicine. Tissue samples (4 mm in diameter) were obtained from primary osteosarcomas, processed, and washed with ice‐cold PBS. Tumor pieces were then dissociated into single cells using Tumor Tissue Digestion Solution (OneTar, China) under gentle shaking at 37 °C for 40 min. After cell counting, the dissociated cells were resuspended in Organoid Culture Matrigel (OneTar, China) and plated as Matrigel domes in 96‐well tissue culture plates. Organoid cultures were maintained at 37 °C with 5% CO_2_, with Organoid Culture Media (OneTar, China) overlaying the Matrigel domes. Organoid growth was monitored weekly. After four days of treatment, live‐dead staining was performed on the organoids. Live cells emitted green fluorescence, whereas dead cells emitted red fluorescence. Fluorescence intensity was analyzed using ImageJ software to calculate the tumor inhibition rate.

### ELISA Assay

The levels of Leptin and A1BG were quantified using the ELISA kits (Jianglai, China). Briefly, 96‐well plates coated with capture antibody were incubated with standards/samples (1:10 dilution) for 2 h at 37 °C. After HRP‐conjugated detection antibody (1:2000) and TMB substrate incubation, absorbance was measured using a microplate reader.

### qPCR Analysis

Total RNA was extracted using TRIzol, reverse‐transcribed into cDNA with PrimeScript RT Master Mix (EZB, China). qPCR was performed using SYBR Green Master Mix (EZB, China) on a LightCycler 480 system (Roche). Cycling conditions: 95 °C/5 min, 40 cycles of 95 °C/10 s, 60 °C/30 s. Relative expression was calculated via ^ΔΔ^Ct method using GAPDH as endogenous control. The Primer sequences were supplied as Table S1 (Supporting Information).

### Statistical Analysis

Statistical analysis was performed using SPSS software (version 22.0; IBM, Armonk, NY, USA). Data are presented as the mean ± standard deviation (SD) from one representative experiment out of three independent experiments, each performed in triplicate. Statistical significance between groups was determined using Student's two‐tailed *t*‐test or the chi‐square test, with a *p*‐value of less than 0.05 considered statistically significant.

## Conflict of Interest

The authors declare no conflict of interest.

## Supporting information



Supporting Information

## Data Availability

The data that support the findings of this study are available from the corresponding author upon reasonable request.
