# Supplementary material for: Adipocytes Promote Cisplatin Resistance through Secreting A1BG and Regulating NAMPT/PARP1 Axis‐Mediated DNA Repair in Osteosarcoma
Source: Adv Sci (Weinh). 2025 Jun 25;12(35):e02926. doi: 10.1002/advs.202502926 (PMC12463075; doi:10.1002/advs.202502926)
Supplement: Supplementary file 1 — Supporting Information [file ADVS-12-e02926-s001.docx]

Supporting Information

**Adipocytes Promote Cisplatin Resistance through Secreting A1BG and Regulating NAMPT/PARP1 axis-mediated DNA Repair in Osteosarcoma**

Yonghui Liang, Zhaohui Li, Lina Tang, Zhen Pan, Xiang Fei, Chen Tan, Aina He*, Qingcheng Yang* and Dongdong Cheng*


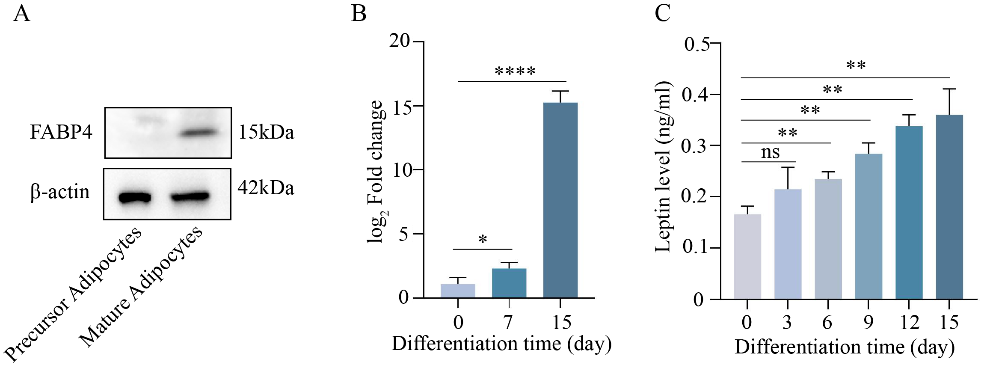


**Figure S1** (A) WB used to detect the FABP4 for precursor and mature 3T3-L1 cells. (B) Leptin mRNA levels in 3T3-L1 cells during differentiation (n=3). (C) Levels of leptin secretion in 3T3-L1 cells during differentiation (n=3). *p<0.05; **p<0.01; ****p<0.0001


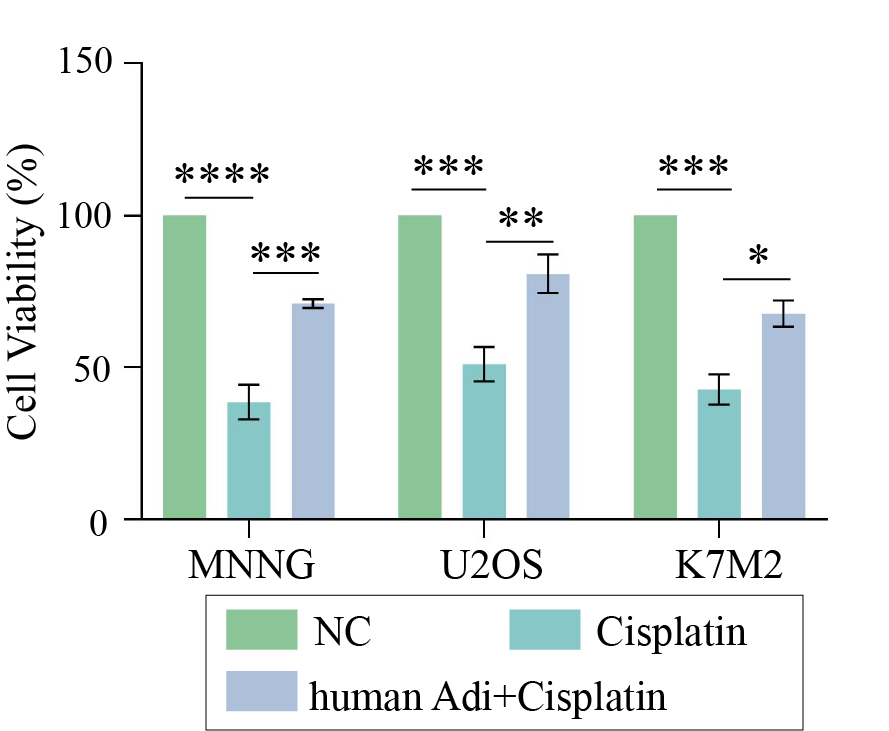


**Figure S2** CCK8 assay for MNNG, U2OS and K7M2 cells with the cisplatin treatment under both control and human Adi-CM conditions (n=3). *p<0.05; **p<0.01; ***p<0.001; ****p<0.0001


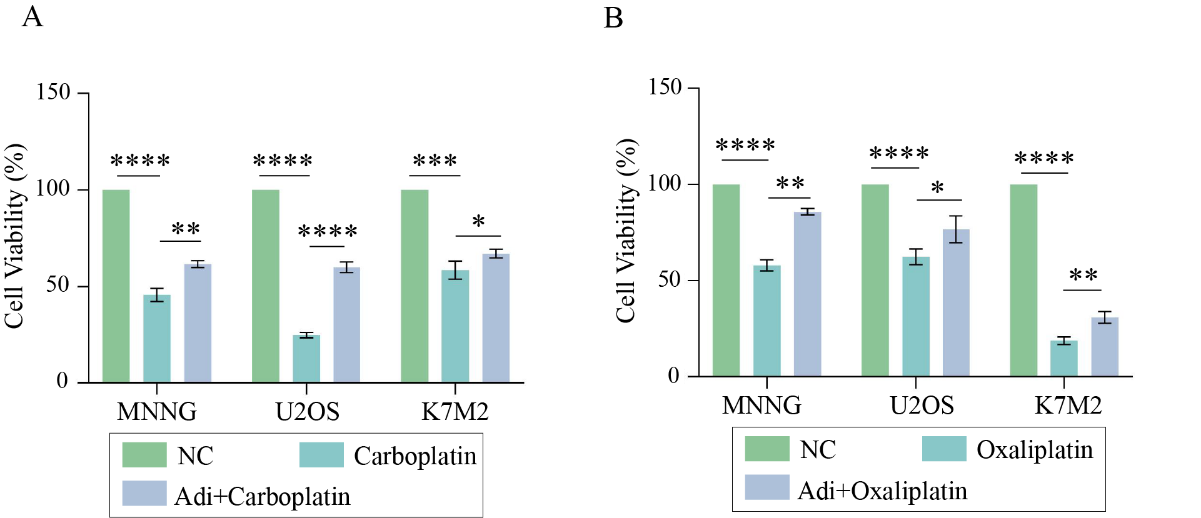


**Figure S3** (A-B) CCK8 assay for MNNG, U2OS and K7M2 cells with the carboplatin and oxaliplatin treatment under both control and Adi-CM conditions (n=3). *p<0.05; **p<0.01; ***p<0.001; ****p<0.0001


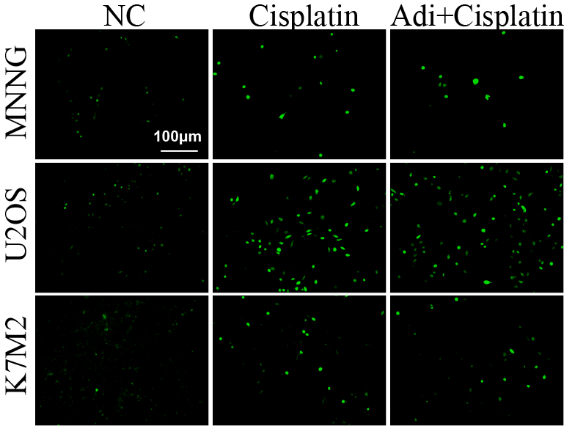


**Figure S4** The ROS level of MNNG, U2OS and K7M2 cells with the cisplatin treatment under both control and Adi-CM conditions.


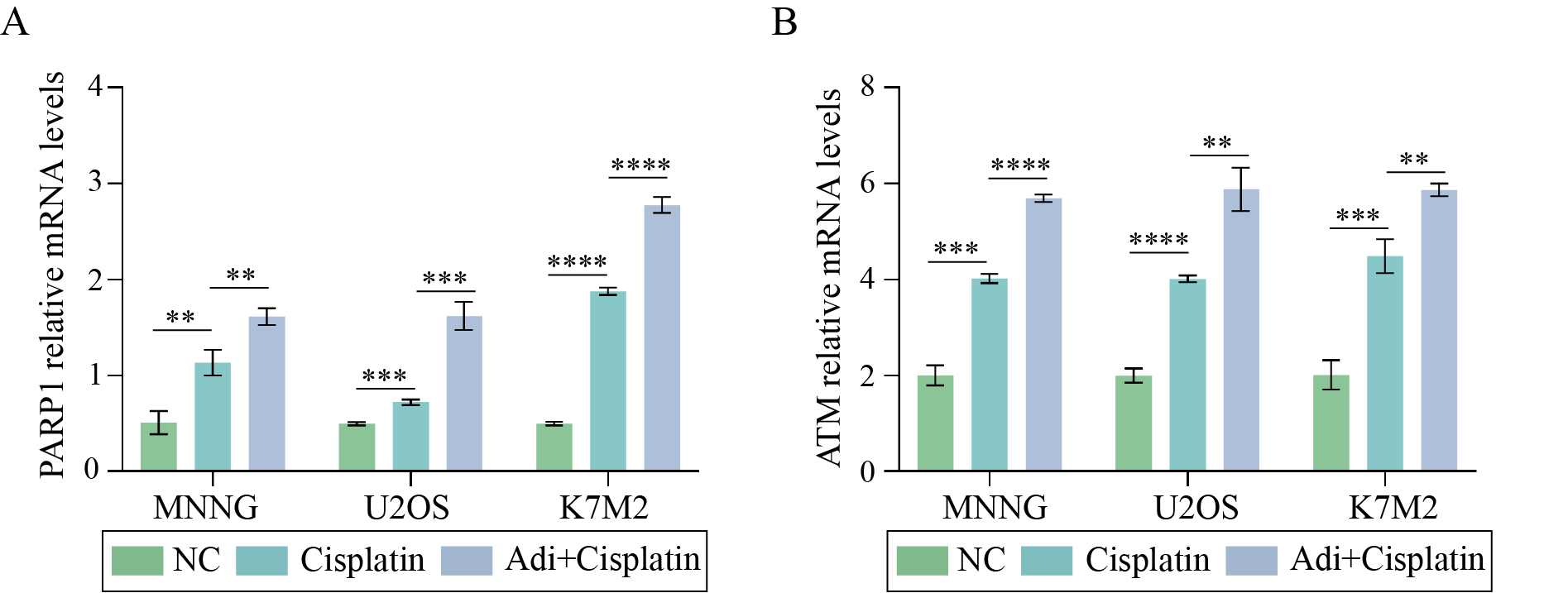


**Figure S5** The transcriptional level of PARP1 and ATM with the cisplatin treatment under both control and Adi-CM conditions in MNNG, U2OS and K7M2 cells (n=3). **p<0.01; ***p<0.001; ****p<0.0001


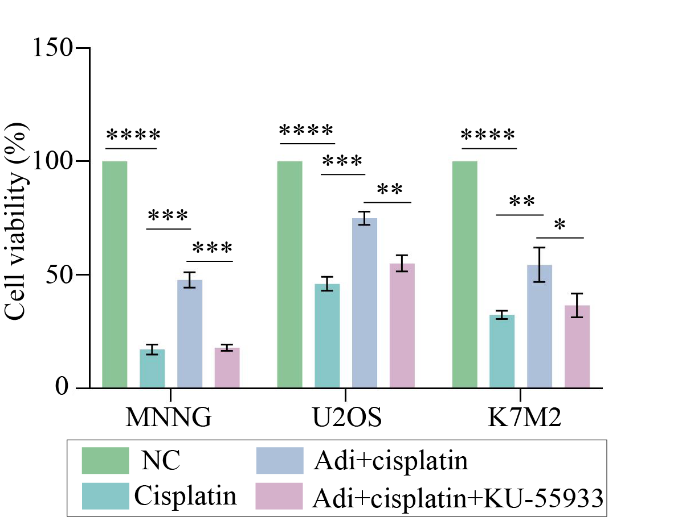


**Figure S6** CCK8 assay for MNNG, U2OS and K7M2 cells under the cisplatin treatment with control, Adi-CM, and the ATM inhibitor Ku-55933(20 μM) conditions for 48 hours (n=3). *p<0.05; **p<0.01; ***p<0.001; ****p<0.0001


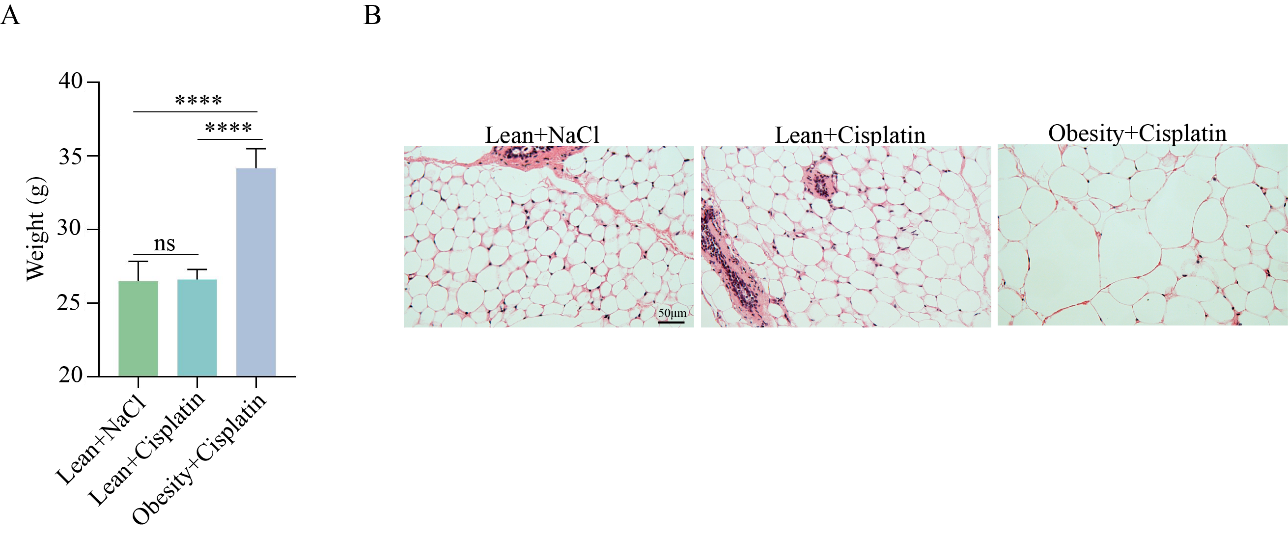


**Figure S7** (A) The weight of normal and obese mice in Figure 2(N) (n=5). (B) The HE staining of the adipose tissue in the normal and obese mice in Figure 2(N). ****p<0.0001


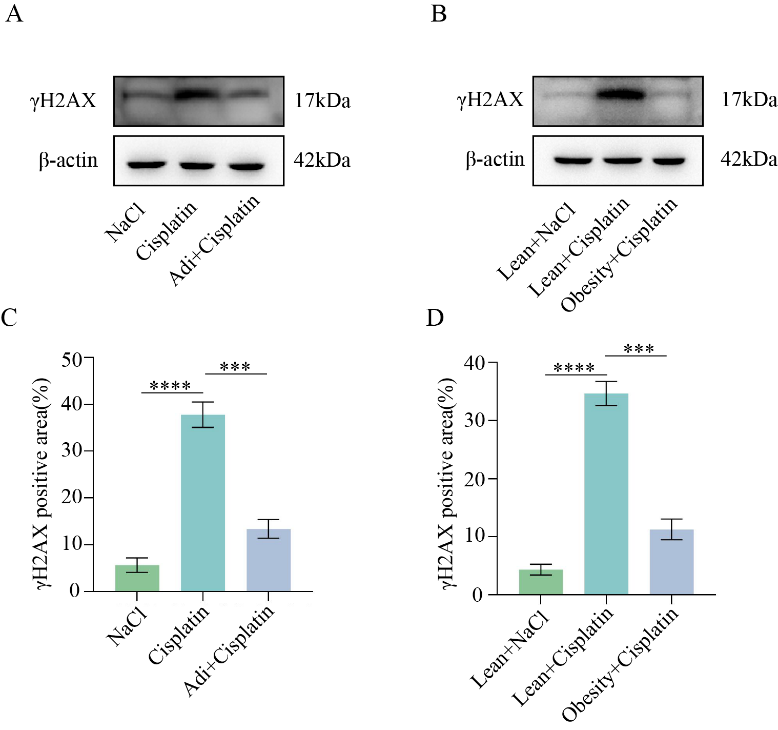


**Figure S8** (A) The Western blot assay was used to detect the γH2AX expression in the xenograft tumors tissues in Figure 2(K). (B) The Western blot assay was used to detect the γH2AX expression in the xenograft tumors tissues in Figure 2(N). (C) The quantitative analysis of γH2AX expression in Figure 2(Q) (n=3). (D) The quantitative analysis of γH2AX expression in Figure 2(R) (n=3). ***p<0.001; ****p<0.0001


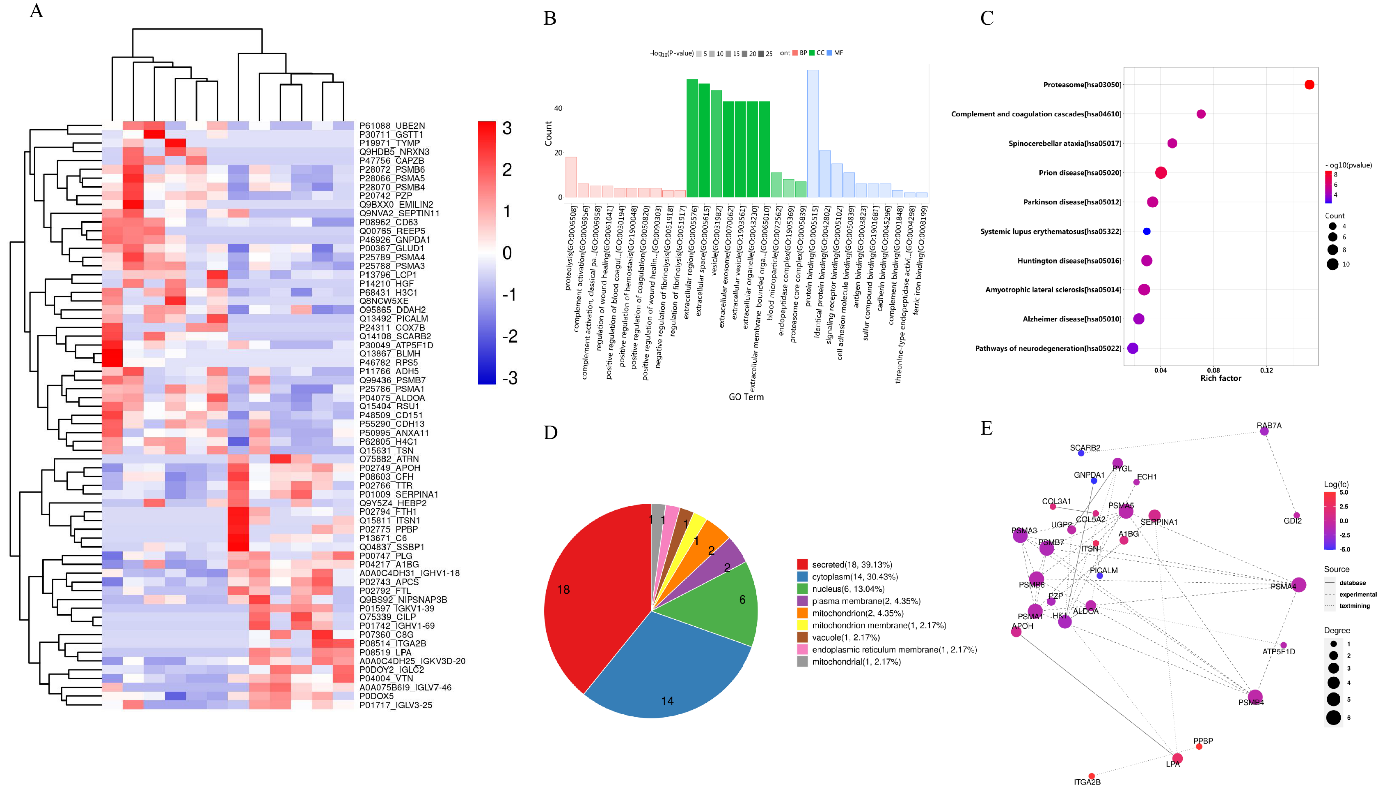


**Figure S9** (A) The heatmap in the secreted protein proteomic analysis. (B) The GO analysis in the secreted protein proteomic analysis. (C) The KEGG analysis in the secreted protein proteomic analysis. (D) The subcellular location analysis in the secreted protein proteomic analysis. (E) The PPI Network analysis in the secreted protein proteomic analysis.


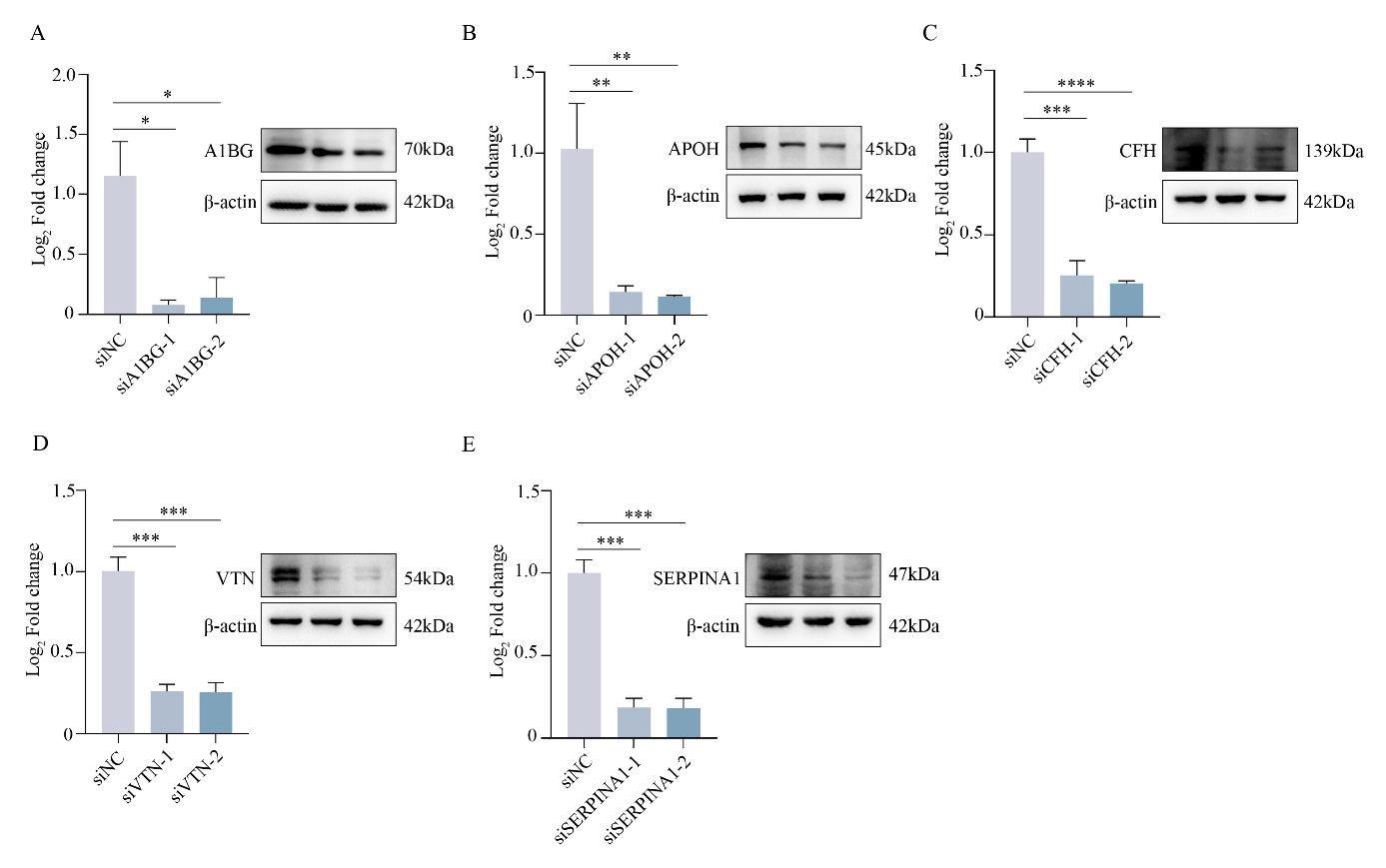


**Figure S10** (A) The qPCR and WB were used to detect the knock down efficiency of A1BG siRNA in 3T3-L1 cells. (B) The qPCR and WB were used to detect the knock down efficiency of APOH siRNA in 3T3-L1 cells. (C) The qPCR and WB were used to detect the knock down efficiency of CFH siRNA in 3T3-L1 cells. (D) The qPCR and WB were used to detect the knock down efficiency of VTN siRNA in 3T3-L1 cells. (E) The qPCR and WB were used to detect the knock down efficiency of SERPINA1 siRNA in 3T3-L1 cells. (n=3) *p<0.05; **p<0.01; ***p<0.001; ****p<0.0001


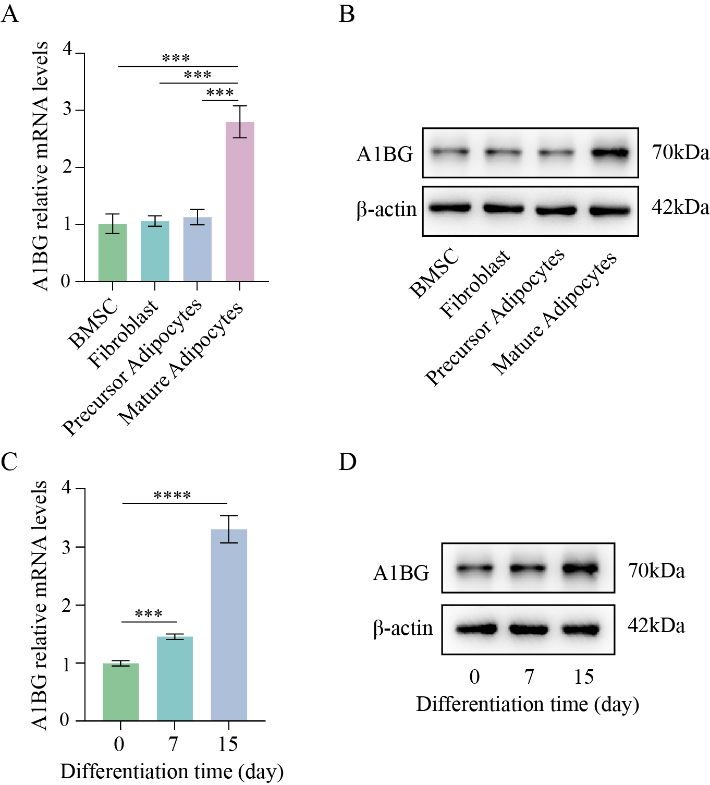


**Figure S11** (A-B) The expression of A1BG in BMSC, fibroblast, precursor adipocytes and mature adipocytes in vivo (n=3). (C-D) The expression of A1BG in 3T3-L1 cells during differentiation (n=3). *p<0.05; **p<0.01; ***p<0.001; ****p<0.0001


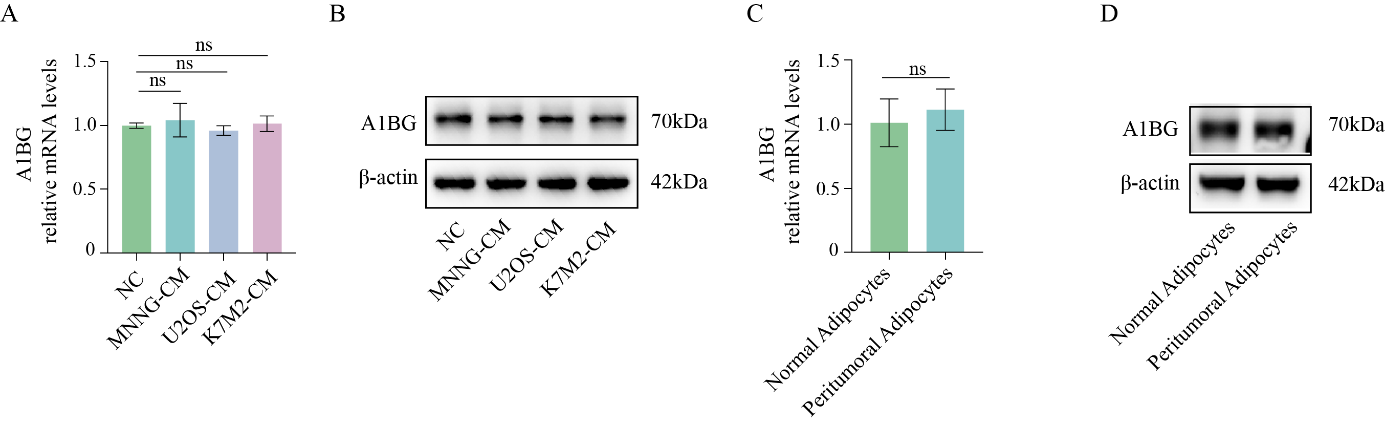


**Figure S12** (A-B) The expression of A1BG under control or MNNG, U2OS and K7M2 condition medium treatment in adipocytes (n=3). (C-D) The expression of A1BG in normal or peritumoral adipocytes (n=3).


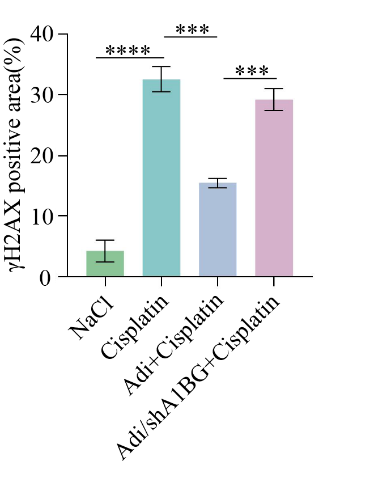


**Figure S13** The quantitative analysis of γH2AX expression in Figure 4(O) (n=3). ***p<0.001; ****p<0.0001


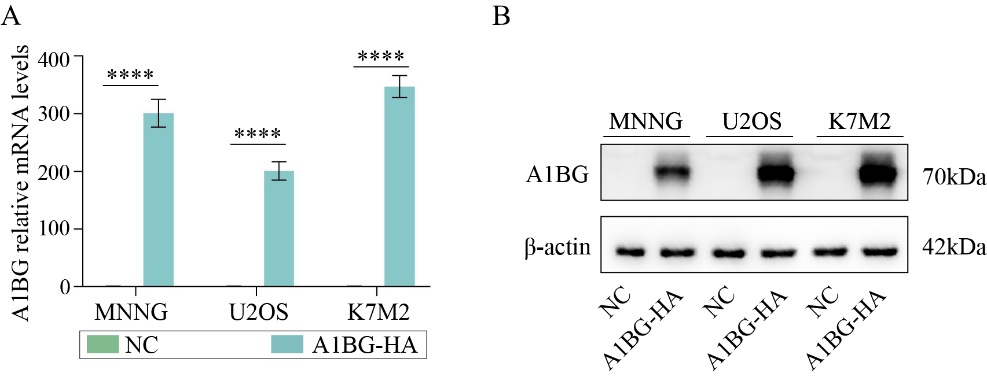


**Figure S14** (A) The qPCR was used to detect the efficiency of over-expression A1BG-HA in MNNG, U2OS and K7M2 cells (n=3). (B) The WB was used to detect the efficiency of over-expression HA-A1BG in MNNG, U2OS and K7M2 cells. ***p<0.001; ****p<0.0001


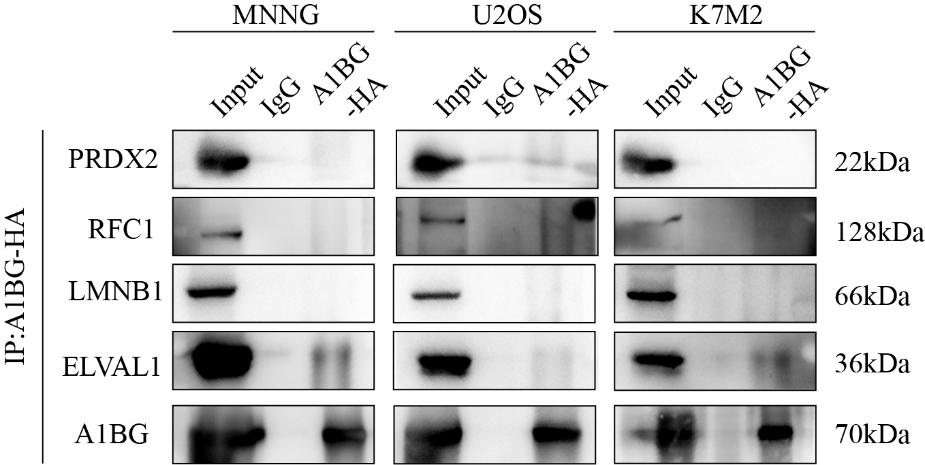


**Figure S15** Co-IP was conducted using an anti-HA antibody. The immunoblotting assay detected anti-A1BG, anti-PRDX2, anti-RFC1, anti-LMNB1 and anti-ELVAL1 antibodies, respectively. IgG was used as a negative control.


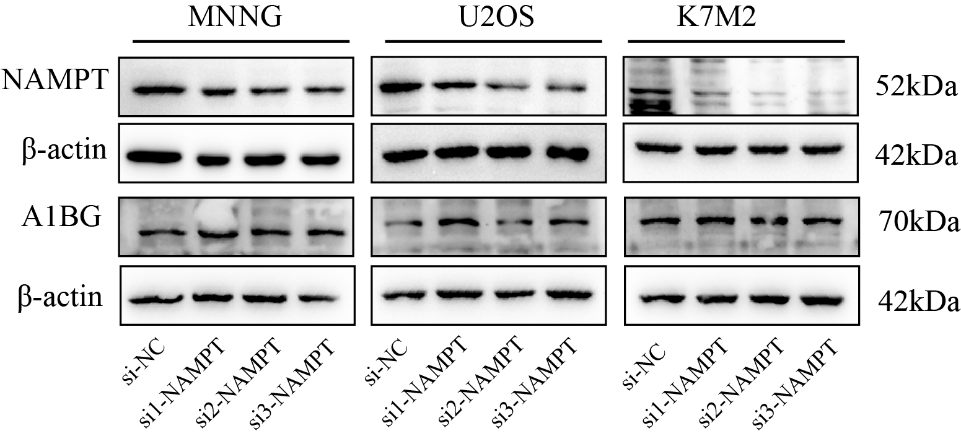


**Figure S16** After NAMPT knockdown, there was no significant decrease observed in A1BG expression at the protein level in MNNG, U2OS and K7M2 cells.


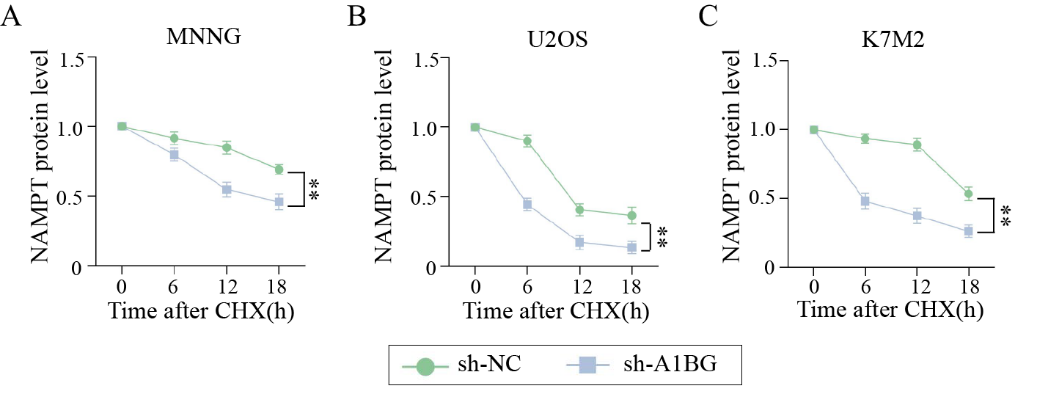


**Figure S17** Quantitative analysis of NAMPT protein levels of CHX chase assays using ImageJ in MNNG (A), U2OS (B) and K7M2 (C) cells.


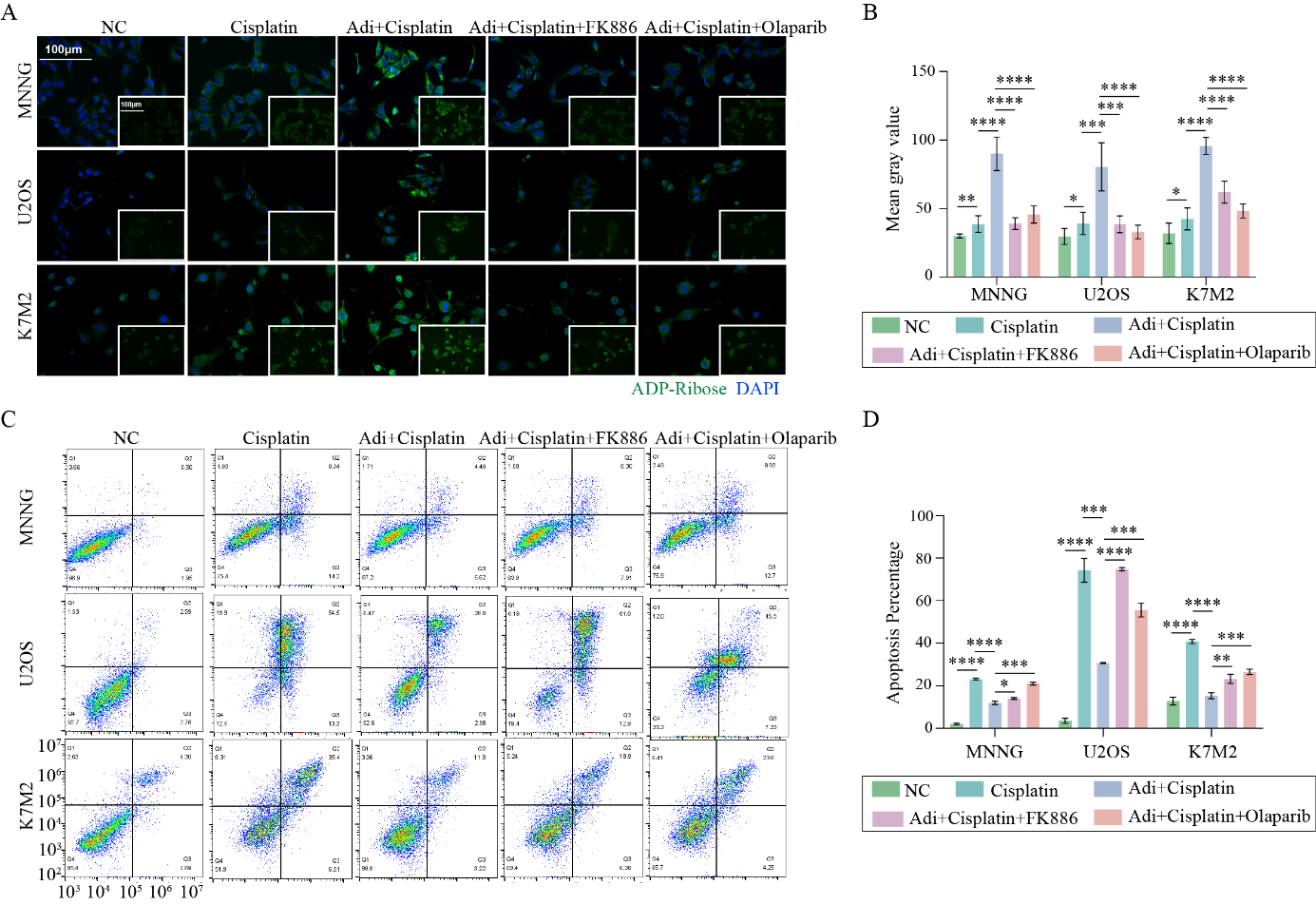


**Figure S18** (A-B) Immunofluorescence analysis of ADP levels in osteosarcoma cells treated with cisplatin under control, Adi-CM, Adi-CM and FK886 (5 nM), and Adi-CM and Olaparib (10 μM) respectively (n=6). (C-D) The apoptosis analysis in osteosarcoma cells with the same treatment in (A) (n=3). **p<0.01; ***p<0.001; ****p<0.0001


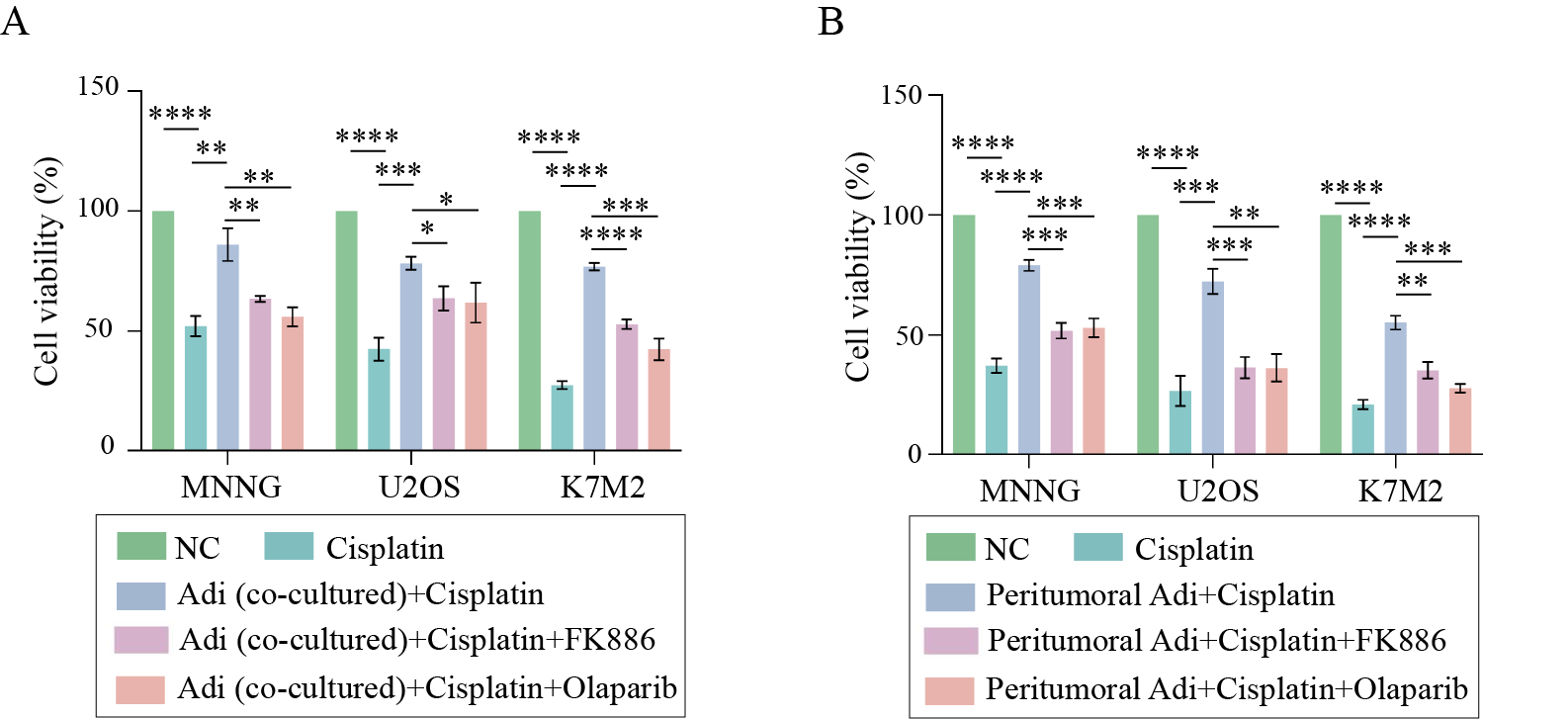


**Figure S19** (A) In a co-cultured system of adipocytes and osteosarcoma, the cell viability of osteosarcoma cells after the cisplatin, cisplatin and FK886, and cisplatin and Olaparib treatment respectively (n=3). (B) The osteosarcoma cells were cultured with the condition medium of peritumoral adipocytes, and the cell viability were tested after the cisplatin, cisplatin and FK886, and cisplatin and Olaparib treatment respectively (n=3). *p<0.05; **p<0.01; ***p<0.001; ****p<0.0001


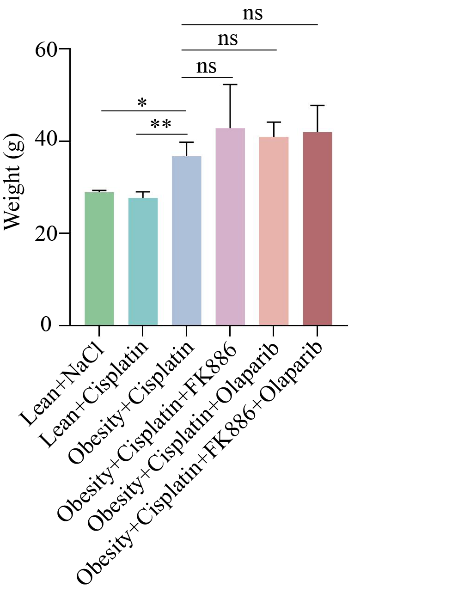


**Figure S20** The weight of the mice in Figure 7(D) (n=4). *p<0.05; **p<0.01


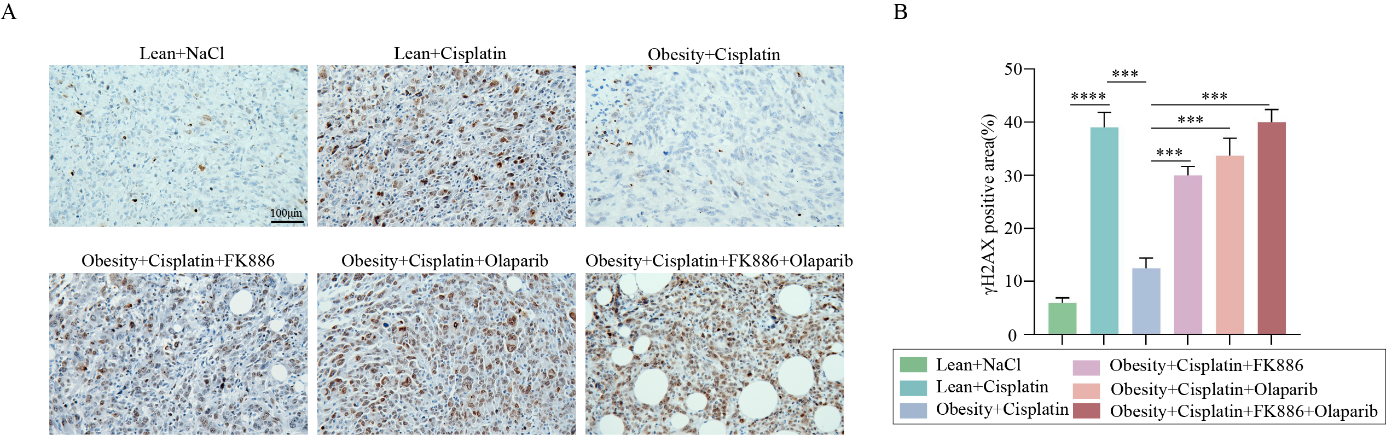


**Figure S21** (A) The γH2AX expression by the IHC assay in the xenograft tumors tissues in Figure 7(D). (B) The quantitative analysis of γH2AX expression in Supplementary Figure 15(A) (n=3). ***p<0.001; ****p<0.0001


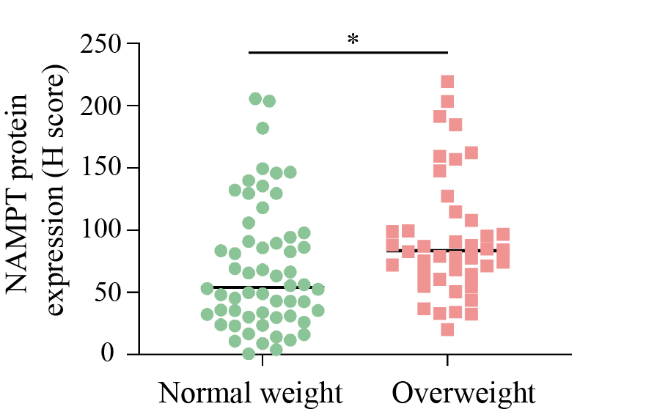


**Figure S22** The IHC analysis (H score) comparing NAMPT expression levels in normal weight (BMI<24) and overweight (BMI≥24) patients. *p<0.05

**Table 1** Primer Sequence

| **Gene- species** | **Forward Primer (5’-3’)** | **Reverse Primer (5’-3’)** |
| --- | --- | --- |
| LEP-mouse | GAGACCCCTGTGTCGGTTC | CTGCGTGTGTGAAATGTCATTG |
| PARP1-human | CGGAGTCTTCGGATAAGCTCT | TTTCCATCAAACATGGGCGAC |
| PARP1-mouse | GGCAGCCTGATGTTGAGGT | GCGTACTCCGCTAAAAAGTCAC |
| ATM-human | ATCTGCTGCCGTCAACTAGAA | GATCTCGAATCAGGCGCTTAAA |
| ATM-mouse | GATCTGCTCATTTGCTGCCG | GTGTGGTGGCTGATACATTTGAT |
| A1BG-mouse | CTCTAATGCTCGATTCTGGCAG | GACCTTAGTCGGCAAATCAACT |
| A1BG-human | TTTTCACGGCATTCAAAGTAGGA | GGCACTTCTGAGGACACCAACA |
| APOH-mouse | CATGTTGCTATTGCAGGACGG | CCCCAGGGTCGTAGGATGT |
| SERPINA1-mouse | AGGCAATGCCACTGCTGTCTTC | GAGATGGACAGTCTGGGGAAGT |
| VTN-mouse | CCCCTGAGGCCCTTTTTCATA | CAAAGCTCGTCACACTGACA |
| CFH-mouse | AGGCTCGTGGTCAGAACAAC | GTTAGACGCCACCCATTTTCC |
| NAMPT-mouse | GCAGAAGCCGAGTTCAACATC | TTTTCACGGCATTCAAAGTAGGA |
| NAMPT-human | CGGCAGAAGCCGAGTTCAA | GCTTGTGTTGGGTGGATATTGTT |
| GADPH-mouse | AGGTCGGTGTGAACGGATTTG | TGTAGACCATGTAGTTGAGGTCA |
| GADPH-human | AATCCCATCACCATCTTCCA | TGGACTCCACGACGTACTCA |
| COL4A1-mouse | ATGGCTTGCCTGGAGAGATAGG | TGGTTGCCCTTTGAGTCCTGGA |
| ADIPOQ-mouse | AGATGGCACTCCTGGAGAGAAG | ACATAAGCGGCTTCTCCAGGCT |
| PLIN1-mouse | GAGAAGGTGGTAGAGTTCCTCC | GTGTGTCGAGAAAGAGTGTTGGC |
| HK2-human | GAGTTTGACCTGGATGTGGTTGC | CCTCCATGTAGCAGGCATTGCT |
| HK2-mouse | CCCTGTGAAGATGTTGCCCACT | CCTTCGCTTGCCATTACGCACG |
| PFKM-human | GCTTCTAGCTCATGTCAGACCC | CCAATCCTCACAGTGGAGCGAA |
| PFKM-mouse | CTGTTCGCTCTACCGTGAGGAT | TTGGAACCACCTTGACCAGTCC |
